# *Haemophilus influenzae* tryptophan biosynthesis is required for lung infection

**DOI:** 10.3389/fcimb.2026.1787089

**Published:** 2026-07-10

**Authors:** Javier Asensio-López, Beatriz Rapún-Araiz, Begoña Euba, Asier Domínguez-San Pedro, Álvaro Sanmartín, Celia Gil-Campillo, David San León, Pablo Chacón, Goizeder Almagro, Ana Ardá, Saioa Burgui, Iñigo Lasa, Alejandro Toledo-Arana, Junkal Garmendia

**Affiliations:** 1 Instituto de Agrobiotecnología, Consejo Superior de Investigaciones Científicas (IdAB-CSIC)-Gobierno de Navarra, Mutilva, Navarra, Spain; 2 Asociación de la Industria Navarra (AIN)-Gobierno de Navarra, Cordovilla, Spain; 3 Centro de Investigación Biomédica en Red de Enfermedades Respiratorias (CIBERES), Madrid, Spain; 4 Laboratory of Microbial Pathogenesis, Navarrabiomed-Universidad Pública de Navarra (UPNA)-Complejo Hospitalario de Navarra (CHN), IdiSNA, Pamplona, Navarra, Spain; 5 Department of Systems Biology, Centro Nacional de Biotecnología, CSIC, Madrid, Spain; 6 Interdisciplinary Platform for Sustainable Plastics towards a Circular Economy-Spanish National Research Council (SusPlast-CSIC), Madrid, Spain; 7 Department of Biological Physical Chemistry, Blas Cabrera Institute of Physical Chemistry, CSIC, Madrid, Spain; 8 Center for Cooperative Research in Biosciences (CIC bioGUNE), Basque Research and Technology Alliance (BRTA), Ikerbasque, Basque Foundation for Science, Bilbao, Spain; 9 Conexión Antimicrobial Resistance-AMR, CSIC, Madrid, Spain

**Keywords:** airway infection, *Haemophilus influenzae*, immune modulation, metabolic drug target, *mtr-sdaCA* excludon, post-transcriptional regulation of gene expression, tryptophan biosynthesis

## Abstract

**Background:**

Tryptophan plays a key role in regulating human lung homeostasis and immunity through the indoleamine 2,3-dioxygenase (IDO) and aryl hydrocarbon receptor (AhR) signalling pathways. In patients with chronic obstructive pulmonary disease (COPD), altered IDO and AhR activity is observed, potentially favouring infection by pathogens capable of synthesizing their own tryptophan. In this study, we investigated the contribution of tryptophan availability to lung infection by *Haemophilus influenzae*, a tryptophan synthesizing pathobiont associated with COPD exacerbations.

**Methods:**

A chemically defined medium with controlled tryptophan levels was developed, and used to determine bacterial (i) metabolite consumption/excretion; (ii) genome-wide differential gene expression and post-transcriptional regulation; (iii) *in vivo* growth in a murine model of lung infection; and (iv) growth upon tryptophan biosynthesis allosteric inhibition.

**Results:**

Under tryptophan-rich conditions, we observed up-regulation of *tnaA* (encoding a tryptophanase) and *tnaB* (encoding a tryptophan transporter), accompanied by down-regulation of tryptophan biosynthetic genes. Furthermore, transcriptomic analysis combined with the ExcludonFinder computational tool generated an excludon map of the *H. influenzae* genome, and identified that the 3´-UTR regions of the convergent *mtr* tryptophan transporter and the *sdaCA* serine transporter-deaminase genes overlap, suggesting a post-transcriptional regulatory link between tryptophan and serine metabolism. *In vivo*, dietary modulation of tryptophan availability in a murine model supported effective lung infection as long as the bacterial tryptophan biosynthetic pathway remains functional. This biosynthetic requirement is supported by the *in vitro* growth inhibitory effect of indole propionic acid, a tryptophan derivative acting as TrpE allosteric inhibitor.

**Conclusions:**

These findings demonstrate that *H. influenzae*’s capacity to synthesize tryptophan is required for infection under host-imposed nutrient limitations, and highlight the potential of tryptophan biosynthesis as a target for antibacterial intervention.

## Introduction

Tryptophan is an essential amino acid that humans cannot synthesize and must obtain through diet. The majority of dietary tryptophan is absorbed in the small intestine and metabolized by the host cells through the kyneurin and serotonin pathways. A smaller fraction reaches the large intestine where it is catabolized by gut commensal bacteria resulting in indole-derived metabolites (Grohmann, 2003; Kałużna-Czaplińska et al., 2019). Tryptophan metabolites act as key signaling molecules that regulate immune responses and maintain homeostasis within the intestinal environment, as well as in distant organs including the lungs (Ma et al., 2020). These metabolites serve as ligands for the aryl hydrocarbon receptor (AhR), a master transcription factor that regulates the expression of the IL-6, IL-10, IL-22, and cytochrome P450s CYPA1 and CYPA2 encoding genes (Masuda et al., 2011; Stockinger et al., 2014; Larigot et al., 2022; Li et al., 2022). The involvement of AhR in pulmonary health and disease is well established, with chronic obstructive pulmonary disease (COPD) representing a prominent example.

COPD is characterized by progressive and irreversible airflow obstruction and includes clinical manifestations such as chronic bronchitis and emphysema. The disease is driven by chronic inflammation, oxidative and nitrosative stress, protease-antiprotease imbalance, and accelerated cell death in the lung tissue (Agustí et al., 2023). Studies have shown that AhR-deficient mice chronically exposed to cigarette smoke develop significant airspace enlargement and concomitant decline in lung function. Similarly, COPD patients exhibit reduced expression of AhR both in lung tissue and systemically (Guerrina et al., 2021). Also related, COPD patients show a progressive reduction in indoleamine 2,3-dioxygenase (IDO) activity, resulting in chronic airway neutrophilic inflammation (Maneechotesuwan et al., 2013). IDO1 catalyzes the rate-limiting step of the kynurenine pathway that converts tryptophan into N-formylkynurenine (Li et al., 2022). Given that IDO1 activity contributes to the generation of AhR ligands, its down-regulation further supports the reduced AhR signaling observed in COPD patients (Puccetti et al., 2018; Guerrina et al., 2021).

The lower airways of COPD patients are frequently colonized by bacterial pathogens. This event promotes airway inflammation, immune system damage, and contributes to disease progression (Luo et al., 2024). Also, COPD patients experience episodes of acute deterioration in respiratory health known as exacerbations, characterized by increased systemic and airway inflammation, worsening of lung function, and a marked deterioration in overall health status. The human pathobiont *Haemophilus influenzae*, particularly in its non-typeable (NTHi) form, is a common colonizer of the COPD lung and one of most frequently isolated bacterial species during exacerbations (Wedzicha and Seemungal, 2007; Hurst et al., 2010; Duell et al., 2016; Su et al., 2018; Ritchie and Wedzicha, 2020; Short et al., 2021). In previous work, we showed that *H. influenzae* lung infection in a murine model down-regulates expression of *ahR* and its downstream effectors *socs2* and *socs4* (Euba et al., 2023). Additionally, *H. influenzae* up-regulates its tryptophan biosynthetic genes during infection of ciliated human bronchial epithelial cells (Baddal et al., 2015), and tryptophan biosynthesis is essential for *H. influenzae* biofilm formation (Harrison et al., 2019).

**Figure 1:**
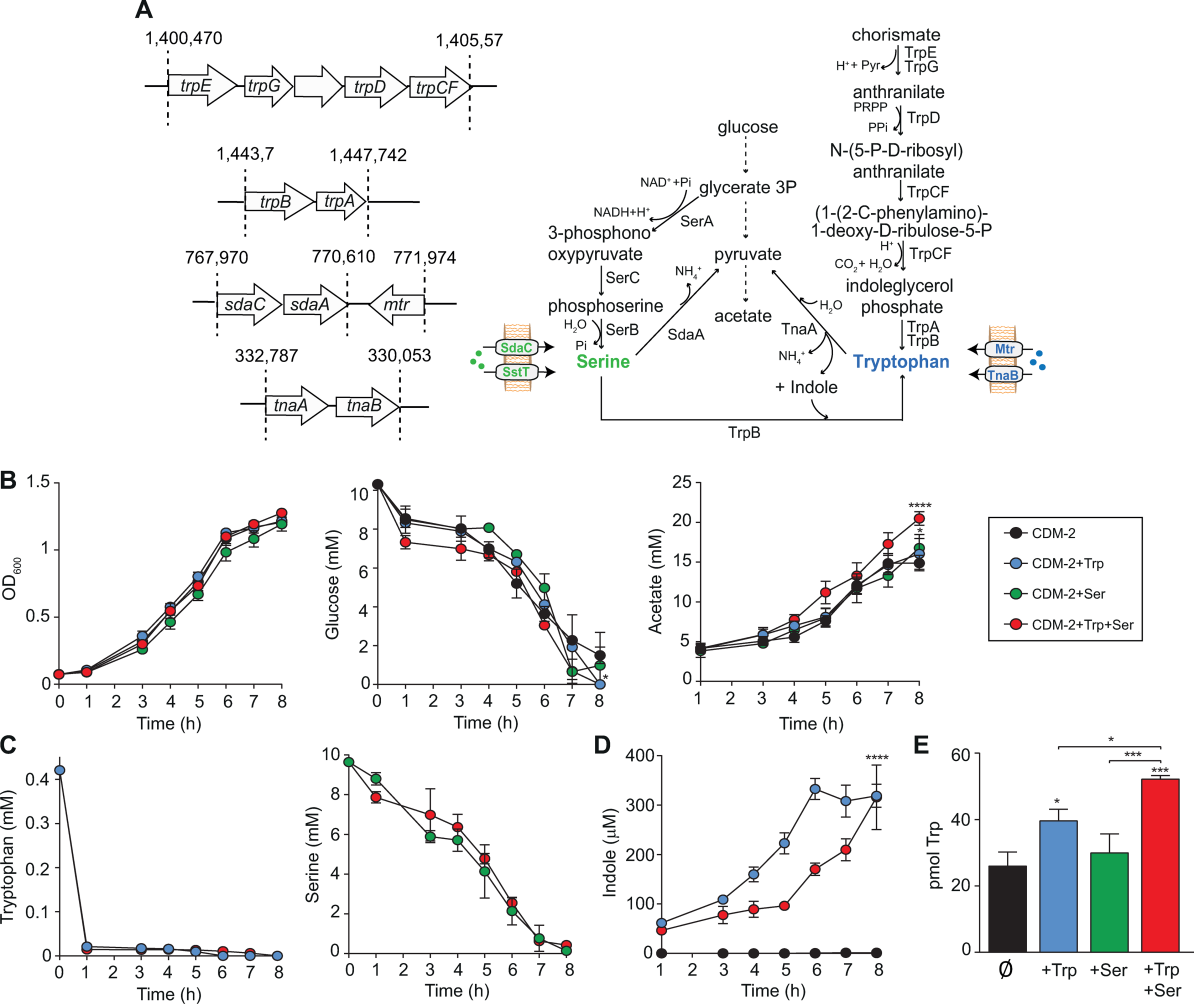
Effects of tryptophan availability on *H. influenzae* metabolism. **(A)** Schematic representation of *H. influenzae* strain NTHi375 tryptophan metabolism. Left panel: genomic organization of loci containing genes involved in tryptophan metabolism and uptake. Right panel: summary of *H. influenzae* tryptophan *de novo* biosynthesis and import. TrpE, anthranilate synthase component I; TrpG, anthranilate synthase component II; TrpD, anthranilate phosphoribosyltransferase; TrpCF, bifunctional indole-3-glycerol-phosphate synthase TrpC/phosphoribosylanthranilate isomerase TrpF; TrpA, tryptophan synthase subunit α; TrpB, tryptophan synthase subunit β; TnaA, tryptophanase; SerA, phosphoglycerate dehydrogenase; SerC, 3-phosphoserine/phosphohydroxythreonine transaminase; SerB, phosphoserine phosphatase; SdaA, L-serine dehydratase; Pi, phosphate; PPi, diphosphate. Color code in panels in **(B–E)**: black, growth in CDM-2; blue, growth in CDM-2 supplemented with tryptophan 0.5 mM; green, CDM-2 supplemented with serine 10 mM; red, CDM-2 supplemented with both tryptophan 0.5 mM and serine 10 mM. At the indicated time points, 1 mL supernatants or bacterial pellets were collected for quantification of OD_600_ and to measure metabolite consumption, excretion or intracellular accumulation levels. **(B)** NTHi375 growth in CDM-2, in the absence or presence of tryptophan 0.5 mM and/or serine 10 mM; OD_600_ was recorded every hour for 8 h (left panel); glucose consumption (middle panel) and acetate excretion (right panel) were determined over time. **(C)** Tryptophan (left panel) and serine (right panel) consumption over time during bacterial growth. **(D)** Quantification of indole excretion over time during bacterial growth. **(E)** Quantification of bacterial intracellular tryptophan levels after 8 h growth. In **(B–E)**, statistical comparisons were performed using two-way ANOVA (Tukey’s multiple comparisons test). Data are shown as mean ± SD (*P<0.05, ***P< 0.001, ****P< 0.0001).

*H. influenzae* synthesizes tryptophan *de novo* using chorismate as a precursor. Moreover, strains containing the *tnaCAB* accessory locus, which encodes the TnaA tryptophanase converting tryptophan into indole and pyruvate, and the TnaB tryptophan transporter (Martin et al., 1998; Munson et al., 2004), can also synthesize tryptophan using serine as a precursor, as the TrpB tryptophan synthase subunit β catalyzes the conversion of indole and serine into tryptophan (Pizer et al., 1969). Besides TnaB, *H. influenzae* incorporates exogenous tryptophan using the Mtr transporter (Harrison et al., 2019), and incorporates exogenous serine using the SdaC and SstT transporters (Kim, 2003; Kim et al., 2003). The convergent *mtr* and *sdaCA* loci present a conserved genomic architecture, which further suggests that tryptophan and serine metabolism may not be only connected biochemically (**Figure 1A**). Taken together, existing evidence led us to hypothesize that tryptophan availability plays a pivotal role in modulating the *H. influenzae*-host airway interaction.

In this study, we analyzed the impact of tryptophan availability, either through exogenous supplementation or *de novo* biosynthesis, on the fitness of *H. influenzae* both *in vitro* and during murine airway infection. Our findings offer novel insights into metabolic regulatory events and into the interplay between host and pathogen, present evidence on *H. influenzae* tryptophan biosynthetic requirement for *in vivo* fitness, and highlight potential metabolic targets for new antimicrobial development.

## Results

### Effect of exogenous tryptophan supplementation on *H. influenzae in vitro* fitness under chemically defined conditions

*H. influenzae* core genome tryptophan biosynthetic genes are organized in the *trpEGDCF* and *trpBA* loci (**Figure 1A**). The *tnaCAB* locus is present in 63.08% of the 325 *H. influenzae* strains whose genomes are complete and publicly available (**Supplementary Dataset S1**). First, we used CDM-2, a defined medium free of tryptophan and serine (Gil-Campillo et al., 2025b) to assess the impact of exogenous tryptophan or serine supplementation on *H. influenzae* fitness. *H. influenzae* strain NTHi375, a representative *tnaCAB*-containing strain, was selected for analysis. The growth of NTHi375 in CDM-2 was comparable regardless of the absence or presence of tryptophan (0.5 mM) and/or serine (10 mM); moreover, glucose consumption and acetate excretion—the main end product of glucose catabolism under aerobic conditions (Othman et al., 2014; López-López et al., 2020) remained unaltered by the addition of these amino acids (**Figure 1B**). When added to the medium, tryptophan and serine were consumed at different rates (faster for tryptophan), which may relate to their respective concentrations, 0.5 mM for tryptophan and 10 mM for serine (**Figure 1C**). Tryptophan supplementation was accompanied by indole excretion to the culture supernatant, measured over time (**Figure 1D**), and by increased intracellular tryptophan concentration, measured at a single time point after 8 h bacterial growth (**Figure 1E**). Simultaneous supplementation with these two amino acids reduced indole excretion compared to that determined when the medium was supplemented with tryptophan only, but increased intracellular tryptophan levels, supporting functional conversion of serine to tryptophan (**Figures 1D, E**).

These findings confirm that CDM-2 supports *H. influenzae* growth through a functional tryptophan biosynthetic pathway. Supplementation with tryptophan promotes its uptake and indole production, likely contributing to increased intracellular tryptophan levels, which are further increased by exogenous serine.

### Antisense regulation on *mtr* exerted by the *sdaCA* 3´-UTR-overlapping mRNA

**Figure 2:**
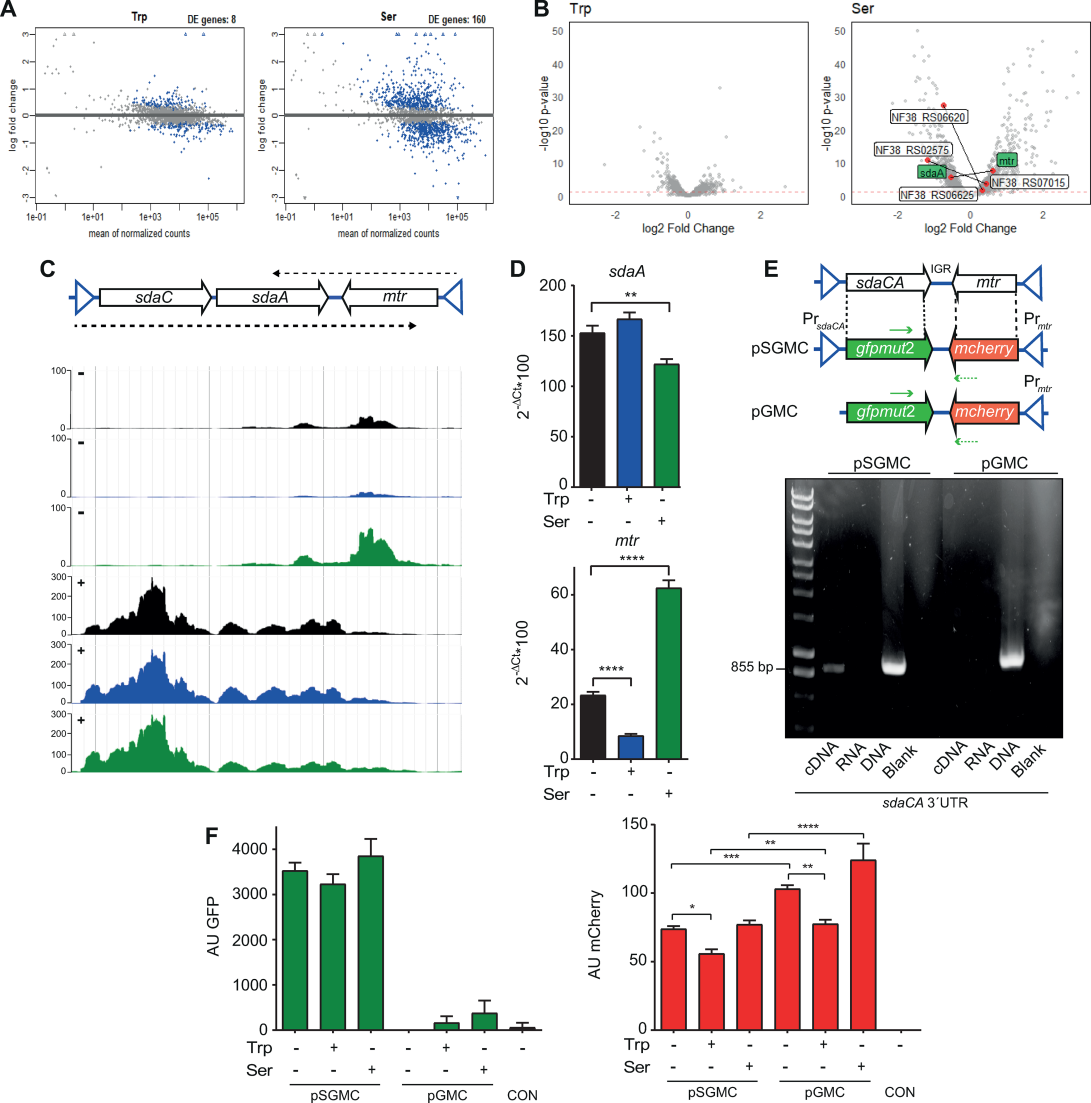
*H. influenzae* genome expression profiling and antisense regulation on *mtr* by *sdaCA* 3´-UTR-overlapping mRNA. **(A)** MA plot showing the relationship between mean expression levels (log_2_ average counts) and log_2_ fold change for all genes in NTHi375 grown in CDM-2 supplemented with tryptophan or serine, compared to CDM-2 alone. Blue dots represent genes with statistically significant differential expression (adjusted P < 0.05). Gene identities are detailed in **Supplementary Dataset S2**. Validation of differentially expressed genes by RT-qPCR on purified RNA is shown in **Supplementary Figure 1**. **(B)** Volcano plot displaying the statistical significance (-log_10_ adjusted P-value) *versus* magnitude of change (log_2_ fold change) for excludon gene pairs under tryptophan (left) and serine (right) supplementation conditions. Pairs of excludon genes with opposite expression patterns are highlighted. Three excludon examples are labelled with gene names when annotated, or locus tags when gene names are not available. **(C)** Architecture and transcriptome analysis of the NTHi375 genome region containing the *mtr* and *sdaAC* loci. Color code: black, CDM-2 grown bacteria; blue, CDM-2 with tryptophan grown bacteria; green, CDM-2 with serine grown bacteria. **(D)** Bacterial cultures were grown in CDM-2 (black), CDM-2+tryptophan (blue) or CDM-2+serine (green). Exponentially grown cultures were collected for RNA extraction and purified RNA was used to determine the ratio of *sdaA* (upper panel) *mtr* (lower panel) gene expression by RT-qPCR. Statistical comparisons of the means were performed with one-way ANOVA (Tukey’s multiple comparisons test). Data are shown as mean ± SD (**P<0.01, ****P<0.0001). **(E)** Fluorescent reporters mimicking the convergent *sdaCA* and *mtr* transcriptional architecture. Antisense regulation exerted by 3´-UTR overlapping mRNA. Agarose gel image shows the products of the RT and subsequent PCR amplification performed for *sdaCA* 3´-UTR in pSGMC and pGMC. Green arrows indicate primers used for *sdaCA* 3´-UTR; dotted arrows indicate primers used for retrotranscription. **(F)** Quantification of GFP and mCherry levels in strains carrying the pSGMC and pGMC plasmids. Bacteria were grown in CDM-2, in the absence or presence of tryptophan or serine. Fluorescence was quantified using excitation wavelengths of 485 and 587 for GFP (left panel) and mCherry (right panel), respectively. Statistical comparisons of the means were performed with a one-way ANOVA (Tukey’s multiple comparisons test). Data are shown as mean ± SD (*P<0.05, **P<0.01, ***P<0.001, ****P<0.0001).

Next, we investigated whether exogenous tryptophan or serine affect gene expression in the NTHi375 strain by genome-wide transcriptomic analysis. We first examined how gene expression changes depending on amino acid availability. Tryptophan led to a limited transcriptional response, mostly consisting of up-regulation of *tnaB* and *tnaA*, which likely reflects the elevated intracellular tryptophan levels and increased indole excretion observed under tryptophan-supplemented conditions, and down-regulated expression of the *trpA* biosynthetic gene (**Supplementary Dataset S2**, sheet 1; **Figure 2A**, left panel; **Supplementary Figure 1**). We also observed down-regulation of *asnA*, which encodes an aspartate ammonia ligase involved in the conversion of aspartate to asparagine, and of *gdhA*, encoding a glutamate dehydrogenase, but no significant differences in the consumption of aspartate, asparagine or glutamate were detected between conditions (**Supplementary Figure 2**). In contrast, exogenous serine induced significant transcriptional changes, revealing a broader network of amino acid-responsive regulatory circuits (**Supplementary Dataset S2**, sheet 2; **Figure 2A**, right panel). Exogenous serine led to up-regulation of the *trpE*, *trpG* and *trpB* tryptophan biosynthetic genes, and of numerous genes encoding products involved in bacterial resistance to nitrogen reactive species and protein stability including proteases and chaperones, which suggests that extracellular serine may be an environmental stressor for *H. influenzae* under the tested conditions.

Determination of genome-wide transcriptomes also enables the mapping of transcript start and end sites, facilitating the identification of potential transcriptional regulatory connections between the untranslated regions (UTRs) of neighboring genes. Contiguous gene pairs that overlap their transcription at the 5´- or 3´-UTRs are part of the so called excludons (Sesto et al., 2013). Excludons mediate post-transcriptional regulation by coordinating the expression of adjacent, often functionally related genes through antisense interactions. Previous identification of *Escherichia coli* excludon pairs participating in several metabolic pathways (Sanmartín et al., 2025), led us to investigate whether this regulatory mechanism contributes to tryptophan metabolism in *H. influenzae*. Using the ExcludonFinder computational tool (Sanmartín et al., 2025), we generated an excludon map of the *H. influenzae* genome. This analysis identified at least 137 convergent excludons (involving overlaps between 3´-UTRs (Menendez-Gil and Toledo-Arana, 2021)) and 40 divergent excludons (overlaps between 5´-UTRs) (**Supplementary Dataset S3**). Among the convergent excludons, several gene pairs were associated with metabolic pathways, including *metQ* (methionine transport) and *glpR* (glucose catabolite repression); *glpX* (fructose metabolism) and *cydC* (gluthatione/cysteine transport); *fadR* (fatty acid metabolism regulator) and *pssA* (phospholipid metabolism). Notably, two convergent excludons involved genes linked to tryptophan metabolism, *trpA* and *asd* (aspartate-semialdehyde dehydrogenase), and the *mtr* and *sdaCA* loci (**Supplementary Dataset S3**; **Figure 1A**). To identify excludons involved in post-transcriptional regulatory events related to tryptophan or serine metabolism, excludon mapping information was combined with differential gene expression analyses from NTHi375 grown in CDM-2 with exogenous tryptophan or serine. We found 13 excludons that displayed inverse expression patterns upon serine supplementation (**Supplementary Dataset S4**). Within the *mtr-sdaCA* excludon, serine supplementation led to increased *mtr* and reduced *sdaCA* expression, which was further confirmed by RT-qPCR (**Figures 2B–D**), consistent with a predicted antagonistic regulatory pattern. This suggests a functional relevance of the excludon architecture in coordinating amino acid transport and metabolism: elevated serine levels may shift gene expression toward enhanced tryptophan import (*mtr*), and reduced serine catabolism (*sdaA* serine deaminase repression).

To validate the regulatory functionality of the *mtr-sdaCA* excludon, we first identified the promoters of *mtr* and *sdaCA* genes by amplifying and cloning their respective putative promoter regions into plasmid pTBH-03, which carries the *gfp* gene as a reporter (Fernández-Calvet et al., 2021; Rapún-Araiz et al., 2023). Following transformation into the heterologous *H. influenzae* RdKW20 strain, bacteria were grown in CDM-2 with Erm_11_, and GFP fluorescence was quantified. Results showed that the *sdaCA* promoter (Pr*_sdaCA_*::*gfp*) exhibit higher activity than the *mtr* promoter (Pr*_mtr_*::*gfp*) (**Supplementary Figure 3**). To analyze how the excludon architecture coordinates the expression of *mtr* and *sdaCA*, a transcriptional reporter plasmid was constructed where only the *mtr* and *sdaCA* open-reading frames (ORFs) were replaced by the *mcherry* and *gfp* ORFs, respectively. Therefore, the native convergent transcriptional architecture, including native promoters, 5´-UTRs, RBSs and intergenic region (IGR) between *mtr* and *sdaA*, were fully preserved generating the pSGMC construct. A second reporter plasmid was constructed to disrupt the excludon transcriptional configuration by deleting the *sdaCA* promoter, which triggers the strongest transcriptional activity in this excludon pair (pGMC). These plasmids were introduced into *H. influenzae* RdKW20, bacteria were grown in CDM-2 with Erm_11_, and overlapping transcription between the *sdaCA* and *mtr* regions was confirmed by oligo-specific PCR using cDNA. A PCR product corresponding to the *sdaCA* transcript was detected within the *mtr* region (**Figure 2E**), indicating transcriptional overlap. To assess the regulatory impact of this overlap, we compared GFP and mCherry fluorescence in the presence (pSGMC) and absence (pGMC) of the *sdaCA* promoter. As expected, removal of the *sdaCA* promoter abolished GFP expression; notably, mCherry fluorescence increased in the absence of the *sdaCA* antisense RNA, suggesting that expression of the *sdaCA* operon negatively regulates *mtr* expression (**Figure 2F**).

Although transcriptomic analysis did not show statistically significant differential expression of *mtr*, a lower transcriptomic signal was observed under tryptophan-supplemented conditions also confirmed by RT-qPCR analysis (**Figures 2C, D**). This aligns with a reduction in both *mtr* transcript levels and mCherry signal when tryptophan was added, indicating that *mtr* expression is responsive to this amino acid availability. In contrast, expression of the *sdaCA-gfp* reporter was unaffected by tryptophan, confirming that the antisense regulation exerted by the *sdaCA* 3′-UTR-overlapping mRNA is independent of tryptophan levels (**Figures 2D, F**). Under serine-supplemented conditions, higher *mtr* transcriptomic and RT-qPCR signals were observed, concomitant to lower RT-qPCR *sdaA* gene expression (**Figure 2D**). GFP and mCherry signals were not altered when serine was added (**Figure 2F**); although unexpected, it could be due to the lack of the *tnaCAB* locus in the RdKW20 heterologous strain genome, which does not couple tryptophan and serine metabolism.

Collectively, the *H. influenzae* excludon map responsive to tryptophan and serine is presented. Our findings reveal a novel layer of regulatory control that links serine and tryptophan metabolism via opposing expression of *mtr* and *sdaCA*. This coordination is mediated by a transcriptional overlap within their 3′-UTRs, enabling a fine-tuned regulatory mechanism responsive to amino acid availability.

### Tryptophan transport is dispensable for *H. influenzae* survival *in vivo*

**Figure 3:**
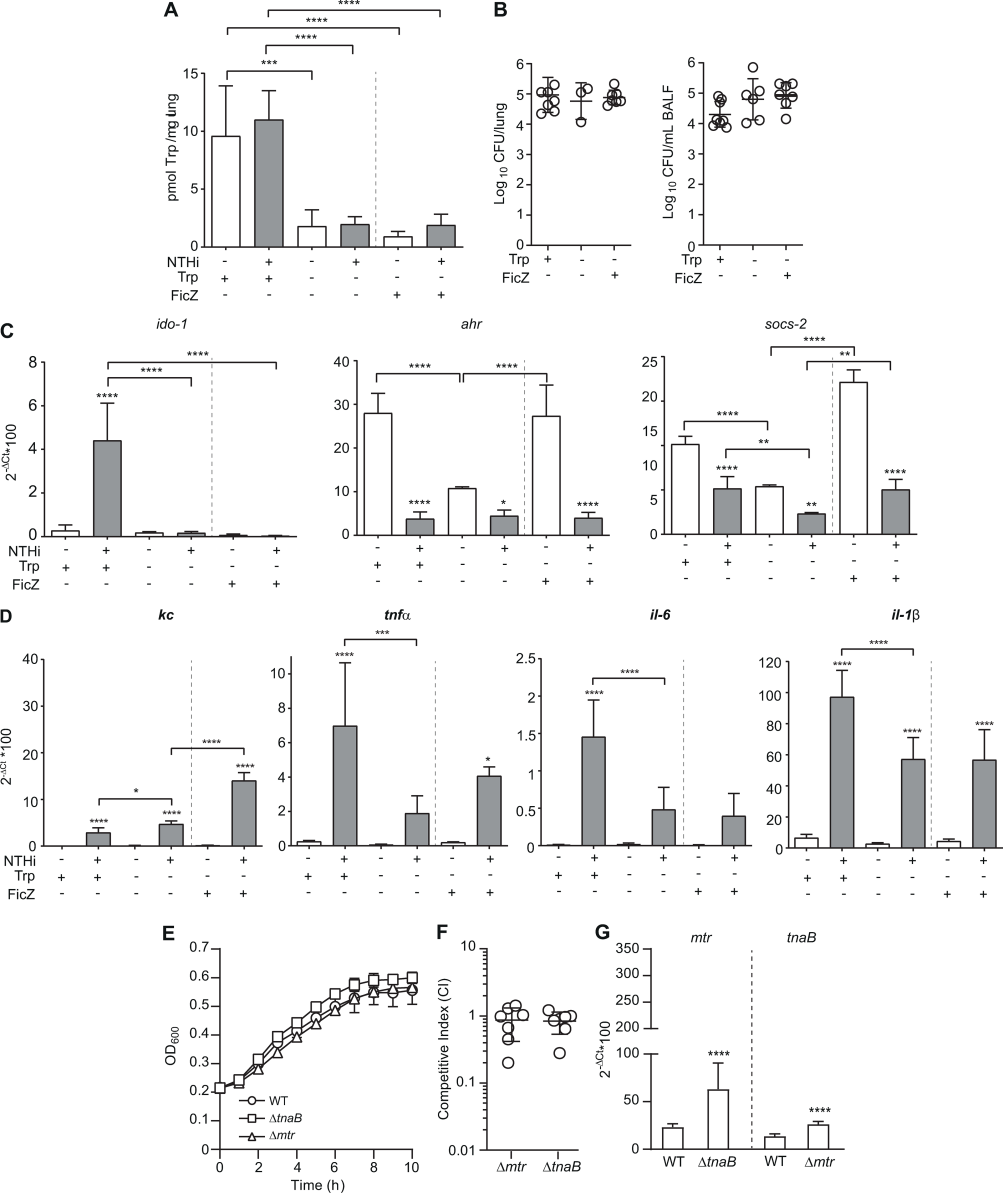
Effects of host tryptophan availability on *H. influenzae* airway infection. **(A–D)** CD1 mice were fed with a standard or tryptophan-depleted diet, administered FICZ when indicated, and infected for 24 h with NTHi375 grown in CDM-2. **(A)** Lung tryptophan was quantified as described in the Methods section. **(B)** Bacterial counts were determined in lungs (log_10_ CFU/lung) and BALF (log_10_ CFU/mL BALF) samples. Host tryptophan availability had no effect on CFU counts. **(C, D)** Lungs were processed for RNA extraction. Relative quantities of mouse *ido-1*, *ahr* and *socs-2* mRNA were measured by RT-qPCR on lung samples **(C)**; relative quantities of mouse *kc*, *tnf-α*, *il-6* or *il-1β* mRNA were measured by RT-qPCR on lung samples **(D)**. In **(A)**, **(C)** and **(D)**, white bars, non-infected animals; grey bars, infected animals; statistical comparisons of the means were performed with a one-way ANOVA (Tukey’s multiple comparisons test). Data are shown as mean ± SD. (*P<0.05, **P<0.01, ***P<0.001, ****P<0.0001). **(E, F)** Effect of inactivating tryptophan transport systems in *H. influenzae in vitro* and *in vivo* fitness. **(E)** Growth of NTHi375 WT, Δ*mtr* and Δ*tnaB* strains in CDM-2. Strains were grown in 96-well plates, OD_600_ was recorded every hour for 10 h. **(F)** Determination of CI. NTHi375 WT, Δ*mtr* and Δ*tnaB* were grown in CDM-2. Mice were infected with bacterial mixed suspensions (WT:mutant, ratio 1:1). Mice were euthanized at 24 hpi, lungs were processed, serially diluted, and plated on sHTM agar, in the presence or absence of antibiotic. WT and mutant CFU counts were used for CI determination. **(G)** Expression of the *mtr* and *tnaB* genes in the NTHi375 WT, Δ*mtr* and Δ*tnaB* strains. Bacterial cultures were grown in CDM-2, exponential cultures were collected for RNA extraction, and purified RNA was used to determine the ratio of gene expression by RT-qPCR. Statistical comparisons of the means were performed with one-way ANOVA (Tukey’s multiple comparisons test). Data are shown as mean ± SD (****P<0.0001).

The observation that *H. influenzae* regulates exogenous tryptophan uptake, as tryptophan supplementation increases *tnaB* expression and decreases *mtr* expression, prompted us to investigate how host tryptophan affects *H. influenzae* lung infection. Two complementary approaches were performed. First, mice were fed either a standard diet (tryptophan+) or a tryptophan-depleted diet (tryptophan−), and then intranasally infected with *H. influenzae* NTHi375 previously cultured in CDM-2. Tryptophan deprivation had a marked physiological impact, as animals on the depleted diet exhibited significant weight lost (standard diet: 31.03 g ± 1.3; tryptophan-depleted diet: 15.38 g ± 0.42), and reduced tryptophan levels were confirmed in lung samples, compared to animals fed with standard diet (**Figure 3A**). Despite this, bacterial counts in both lung and bronchoalveolar lavage fluid (BALF) samples were comparable between the two groups (**Figure 3B**). Induction of host IDO is a nascent strategy to starve pathogens of tryptophan, as IDO1 activity catabolizes this essential amino acid (Gautam et al., 2018; Li et al., 2022). We observed increased *ido-1* expression in the lungs of *H. influenzae*-infected mice fed with a standard diet (**Figure 3C**, left panel). Since IDO activity produces ligands for AhR, up-regulation of *ido-1* could enhance AhR signaling during infection. However, consistent with our previous transcriptomic analysis (Euba et al., 2023), we now confirm that *ahr* expression is reduced in *H. influenzae* infected lungs. Moreover, dietary tryptophan deprivation further decreases *ahr* expression—likely due to reduced availability of AhR ligands, and such reduction is exacerbated by infection (**Figure 3C**, middle panel). A similar trend was observed for the downstream AhR effector *socs2* (**Figure 3C**, right panel). Next, we aimed to uncouple tryptophan availability from AhR signaling by administering the AhR ligand 6-formylindolo[3,2-b]carbazole (FICZ) to Trp-deprived mice. In the absence of infection, FICZ boosted AhR activity; upon infection, bacterial counts were maintained, together with decreased *ahR* and *socs2* gene expression (**Figure 3C**, middle and right panels). Thus, infection may weaken AhR immunomodulatory effects which, as a consequence, could enhance pro-inflammatory responses (Euba et al., 2023). Indeed, NTHi375 infection resulted in up-regulation of the *kc*, *tnf-α*, *il-6* and *il-1β* pro-inflammatory genes, compared to non-infected animals, and this response was observed regardless of dietary tryptophan availability (**Figure 3D**).

Secondly, we assessed the effect of inactivating tryptophan transport on *H. influenzae* infection. Inactivation of the *mtr* or the *tnaB* genes had no impact on bacterial growth in CDM-2 (**Figure 3E**). When animals fed with standard diet were infected with those mutants previously cultured in CDM-2, and competitive index (CI) was determined by co-infection with the NTHi375 wild-type (WT) strain (Gil-Campillo et al., 2025a), *in vivo* fitness was unaltered (**Figure 3F**). Therefore, exogenous tryptophan does not seem to be critical for lung infection. This may be also explained by compensatory mechanisms, as transport gene inactivation resulted in increased expression of the alternative transporter (**Figure 3G**).

Together, these results showed that dietary tryptophan does not influence bacterial counts within the lung, its uptake is dispensable likely due to the bacterial ability to synthesize its own tryptophan, and infection modulates host lung responses to infection as evidenced by monitoring *ido-1* gene expression. Indeed, IDO and AhR signaling seem to be uncoupled in response to *H. influenzae* infection (*ido-1* expression, up-regulated; *ahr* expression, down-regulated), despite IDO activity likely providing AhR ligands.

### *De novo* tryptophan biosynthesis is required for *H. influenzae* survival in a murine lung infection model

**Figure 4:**
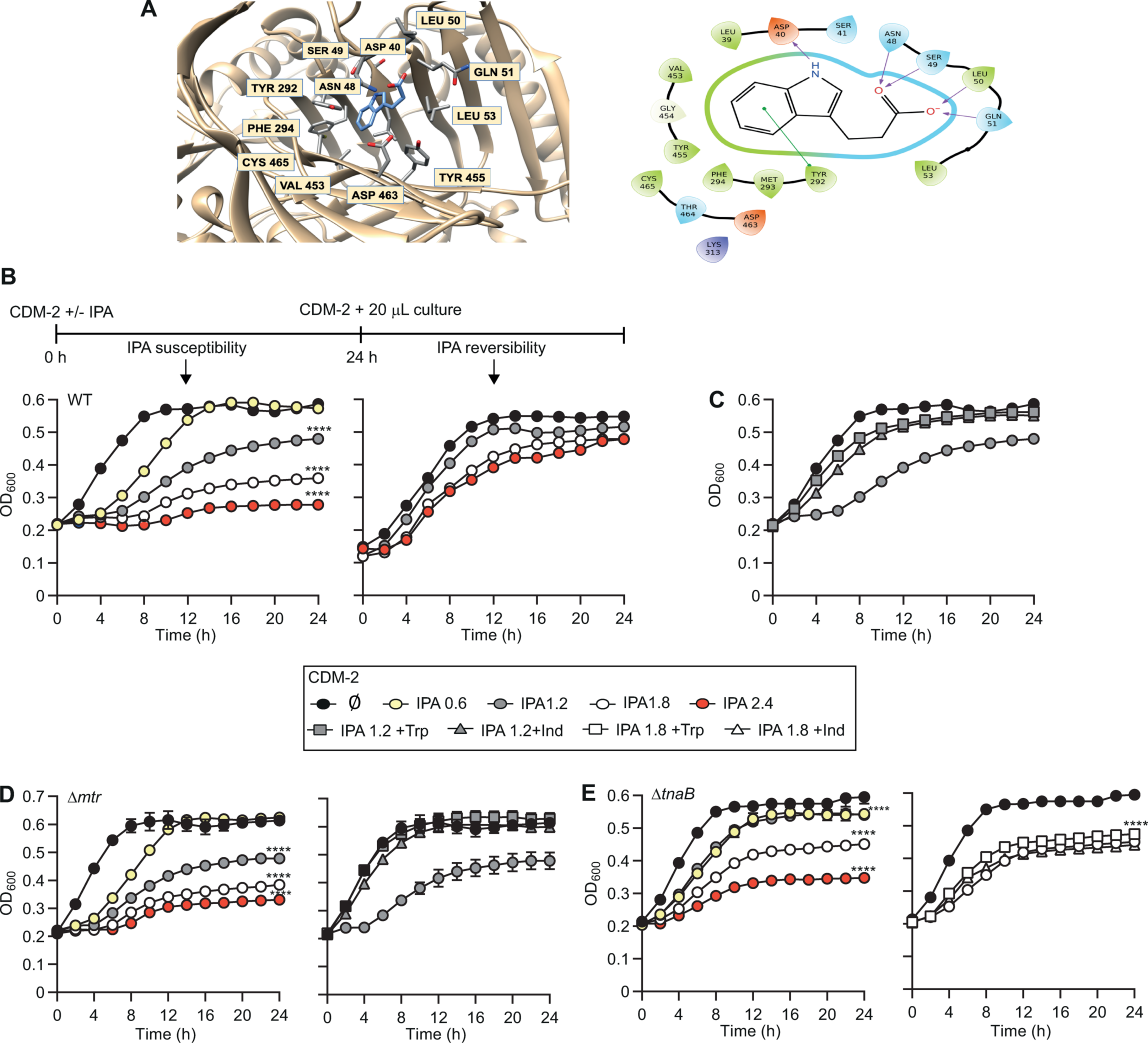
Indole propionic acid (IPA) inhibits NTHi375 *in vitro* growth. **(A)** Left panel: predicted binding site of IPA in the structure of TrpE_NTHi375_ (AF2 model), using Glide; specific amino acid residues involved in binding are labelled. Right panel: 2D interaction diagram of IPA bound to TrpE generated using the Ligand Interaction Diagram Panel of Maestro (Schrödinger Release 2022-2). Hydrophobic residues are shown in green; polar residues in cyan; positively charged residues in purple; and negatively charged residues in orange. Hydrogen bonds are represented by purple arrows; red lines indicate π-cation interactions. For **(B–E)**, symbol legend is indicated in the inset mid panel. **(B)** Analysis of IPA effect on NTHi375 WT growth. Left panel, susceptibility assay: tested IPA concentrations ranged from 0.6 to 2.4 mM. Inhibitory effects were shown for IPA 1.2, 1.8 and 2.4 mM, from 2 to 24 h (****P<0.0001). IPA 0.6 mM showed inhibitory effect from 2 to 12 h (P<0.01). Right panel, reversibility assay: a 20 µL aliquot of each IPA containing culture used for the susceptibility assay was added to fresh CDM-2 and OD_600_ was measured for 24 additional hours. **(C)** IPA effect restoration assay: restoration of IPA 1.2 mM inhibitory effect by tryptophan or indole addition. **(D)** Analysis of IPA effect on NTHi375Δ*mtr* growth. Inhibitory effects are shown for IPA 1.2, 1.8 and 2.4 mM, from 2 to 24 h (****P<0.0001); IPA 0.6 mM showed inhibitory effect from 2 to 12 h (P<0.01) (left panel). Restoration of IPA 1.2 mM inhibitory effect by tryptophan or indole supplementation is shown in the right panel. **(E)** Analysis of IPA effect on NTHi375Δ*tnaB* growth. Significant differences were observed at all tested concentrations from 2 to 24 h (****P<0.0001) (left panel). IPA 1.8 mM inhibitory effect was not restored by tryptophan or indole supplementation (right panel). In all cases, statistical comparisons of the means were performed with two-way ANOVA (Tukey’s multiple comparisons test).

Given that *H. influenzae* infection up-regulates *ido-1* expression, we next hypothesized that bacterial *de novo* tryptophan synthesis may be an adaptive trait for survival within the host during tryptophan catabolism by IDO. To evaluate this hypothesis, we used two complementary strategies to inhibit *H. influenzae* tryptophan biosynthesis, by the means of pharmacological or genetic inhibitions. First, we explored whether pharmacological inhibition of tryptophan biosynthesis affects *H. influenzae* growth in CDM-2 medium. Previous work showed that indole propionic acid (IPA) inhibits the growth of *Mycobacterium tuberculosis* by acting as an allosteric inhibitor of TrpE, the enzyme catalyzing the conversion of chorismate to anthranilate in the tryptophan biosynthetic pathway (**Figure 1A**) (Negatu et al., 2018; Negatu et al., 2019). IPA is a tryptophan-derived metabolite produced by gut microbiota commensal bacteria such as *Clostridium sporogenes* (Dodd et al., 2017; Agus et al., 2018). To investigate whether a similar mechanism applies to *H. influenzae*, we used AlphaFold2 (Jumper et al., 2021) to predict the structure of TrpE from the NTHi375 strain, and Glide docking (Friesner et al., 2004) to model the binding mode of IPA to TrpE (**Figure 4A**, left panel; **Supplementary Figure 4**). A 2D schematic of the ligand–protein interactions from the best docking pose is also shown (**Figure 4A**, right panel). The predicted binding includes interactions involving the carboxyl group, with residues Ser49, Asn48, Leu50, and Gln51 (hydrogen bonding), as well as interactions with the indole ring, involving residues Tyr292 (π–π interaction), Val453, Tyr455, Phe294 (hydrophobic interactions), and Asp40 (electrostatic interaction). Most of these interactions have been experimentally observed in the crystallographic structure of *Serratia marcescens* TrpE bound to an L-tryptophan inhibitor (Spraggon et al., 2001). Correlation between specific L-tryptophan and IPA-amino acid interactions is evident when comparing binding sites (**Figure 4A**; **Supplementary Figure 4**). The indole ring of the experimental and predicted molecules occupies almost the same position in the binding cavity, suggesting that IPA could act as an allosteric inhibitor of *H. influenzae* TrpE.

To assess growth inhibition by IPA, we performed susceptibility assays using a concentration range of 0.6 to 2.4 mM. IPA inhibited the growth of the NTHi375 strain in a dose-dependent manner (**Figure 4B**, left panel). This inhibitory effect was reversible, as IPA-treated cultures regained growth when transferred to fresh CDM-2 medium (**Figure 4B**, right panel). Likewise, inhibition was abolished when exogenous tryptophan or indole were added (**Figure 4C**). We also examined whether the Mtr and TnaB tryptophan transporters contribute to the observed IPA effect. Both NTHi375Δ*mtr* and Δ*tnaB* strains showed dose-dependent growth inhibition by IPA (**Figures 4D, E**, left panels). Exogenous tryptophan or indole rescued the IPA effect in the Δ*mtr* strain (**Figure 4D**, right panel), but not in the Δ*tnaB* mutant (**Figure 4E**, right panel). This suggests that TnaB may play a predominant role in tryptophan/indole uptake, consistent with increased *tnaB* and decreased *mtr* expression upon tryptophan supplementation, and not compensated by the above shown increased *mtr* gene expression upon *tnaB* inactivation (see **Figure 3G**). As a result, IPA inhibits *H. influenzae* growth in a dose-dependent and reversible manner. These results prompted us to hypothesize that IPA may have an inhibitory effect *in vivo*. We sought to determine the effect of IPA oral administration *in vivo* by NTHi respiratory infection of mice. We used (i) a regimen of oral IPA (50 or 100 mg/kg) consisting of daily administrations during 4 days prior to infection, and (ii) a regimen of oral IPA (150 mg/kg) consisting of daily administrations during 3 days prior to- and 1 day post-infection. Significant differences in terms of bacterial counts between control untreated- and treated animals were not observed (**Supplementary Figure 5**). A similar trend was observed for *M. tuberculosis*, as IPA *in vitro* inhibitory effects did not translate to reduced lung bacterial load in a mouse model of acute *M. tuberculosis* infection (Negatu et al., 2018).

**Figure 5:**
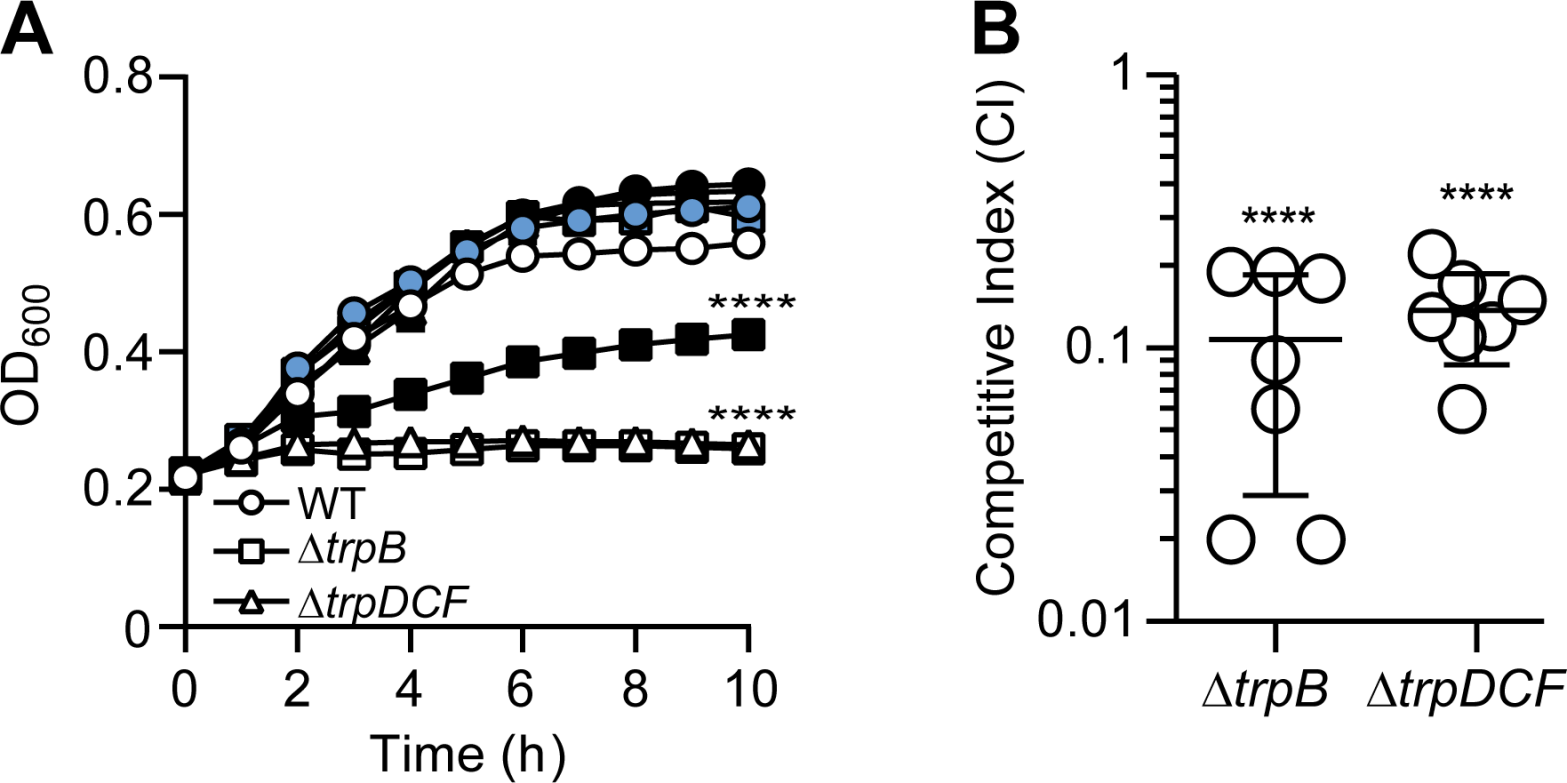
Effect of tryptophan biosynthesis on *H. influenzae* airway infection. **(A)** Growth of NTHi375 WT, Δ*trpB* and Δ*trpDCF* strains in CDM-2, in the absence (white symbols) or presence of tryptophan 0.5 mM (blue symbols) or indole 0.5 mM (black symbols). Strains were grown in 96-well plates, OD_600_ was recorded every hour for 10 h. Statistical comparisons of the means were performed with two-way ANOVA (Tukey’s multiple comparisons test). NTHi375Δ*trpB* and Δ*trpDCF* mutants showed no growth in CDM-2 (from 2 to 10 h, ****P<0.0001). NTHi375Δ*trpB* growth was fully or partially restored by tryptophan and indole, respectively (from 3 to 10 h, ****P<0.0001). NTHi375Δ*trpDCF* growth was restored in both cases, by addition of either tryptophan or indole. **(B)** Determination of competitive index (CI). NTHi375 WT, Δ*trpB* and Δ*trpDCF*, were grown in CDM-2 with tryptophan. CD1 mice were infected with bacterial mixed suspensions (WT:mutant, ratio 1:1). Mice were euthanized at 24 hpi, lungs were processed, serially diluted, and plated on sHTM agar, in the presence or absence of antibiotic. WT and mutant CFU counts were used for CI determination. Statistical comparisons of the means were performed with an unpaired t-test. Data are shown as mean ± SD (****P<0.0001).

Secondly, we generated the NTHi375Δ*trpB* and Δ*trpDCF* mutant strains to genetically inhibit *de novo* tryptophan biosynthesis. TrpB is involved in the last steps of tryptophan synthesis, converting either indoleglycerol phosphate, or indole and serine, to tryptophan. TrpD (anthranilate phosphoribosyltransferase) and TrpCF (bifunctional indole-3-glycerol-phosphate synthase) are involved in the first steps of tryptophan biosynthesis, producing indoleglycerol phosphate (**Figure 1A**). Bacterial growth assays in CDM-2 confirmed that both mutants are auxotrophs for tryptophan, and their growth was restored by tryptophan supplementation. Indole addition also restored growth of Δ*trpDCF* mutant, and partially restored growth of the Δ*trpB* strain, suggesting that TrpB may not be the only enzyme catalyzing serine and indole conversion to tryptophan (**Figure 5A**). Strains were grown in CDM-2 supplemented with tryptophan, and CI was determined in a murine model of lung infection. Both auxotrophic mutants exhibited significantly reduced fitness *in vivo*: NTHi375Δ*trpB* (CI = 0.11 ± 0.08) and Δ*trpDCF* (CI = 0.14 ± 0.05) (**Figure 5B**). These findings demonstrate that *de novo* tryptophan biosynthesis is essential for *H. influenzae* survival in the murine airway.

## Discussion

Access to nutrients is essential for pathogen survival within the host. While humans get their tryptophan supply through the diet (Kałużna-Czaplińska et al., 2019), pathogenic bacteria often not only import but also synthesize their own tryptophan *de novo*, which is considered an adaptation for within host pathogen survival during tryptophan catabolism by IDO. *H. influenzae* synthetizes tryptophan from both chorismate, or serine and indole, and imports it via the Mtr and TnaB transporters (Harrison et al., 2019). Here, we provide evidence that *H. influenzae* requires *de novo* tryptophan biosynthesis to maintain its fitness within the murine lung. This requirement appears to be linked, through currently unknown mechanisms, to down-regulation of AhR signaling, despite concurrent up-regulation of IDO.

Establishing *ad hoc* bacterial growth conditions for this study was key, as the previously used chemically defined medium (CDM) is based on RPMI 1640 and contains tryptophan (López-López et al., 2020). Here, we minimized medium complexity while obtaining reproducible bacterial growth, achieved by adding all amino acids except tryptophan and serine. When grown in CDM-2, bacteria have to synthesize their own tryptophan and serine, being the later also a precursor for tryptophan biosynthesis by strains containing the *tnaCAB* locus. Indeed, lack (CDM-2) or lower detection (CDM-2 supplemented with serine, compared to tryptophan supplementation) of excreted indole may relate to indole channeling to tryptophan conversion. Under the conditions tested, the effect of exogenous tryptophan availability and uptake was subtle in terms of *in vitro* bacterial growth, transcriptomic profiling and *in vivo* bacterial fitness. However, our study design revealed novel information relevant to our understanding about the *H. influenzae*-host interplay. We show that tryptophan auxotrophy limits *in vivo* fitness by causing attenuation in a murine model of lung infection. Thus, tryptophan *de novo* biosynthesis is required for infection. Unfortunately, we do not provide evidence for genetic complementation of the Δ*trpB* and Δ*trpDCF* mutants in this study. Linear DNA cassettes for mutant complementation were generated, but recombinant complemented variants could not be obtained after a significant number of trials. Aiming to reinforce our CDM-2 observations, the NTHi375Δ*trpB* and Δ*trpDCF* mutant strains were grown in sBHI, which contains tryptophan 0.288 mM, and used for CI determination, also showing reduced fitness *in vivo*, NTHi375Δ*trpB* (CI = 0.17 ± 0.16) and Δ*trpDCF* (CI = 0.13 ± 0.24), which supports that *de novo* tryptophan biosynthesis is needed for *H. influenzae* survival in the murine airway.

We also present novel information on excludon-mediated regulation in *H. influenzae*. Transcriptomic analyses show that contiguous bacterial genes often exhibit overlapping transcription, forming structures known as excludons (Sáenz-Lahoya et al., 2019; Toledo-Arana and Lasa, 2020; Menendez-Gil and Toledo-Arana, 2021). This architecture enables inverse coordination of gene expression, whereby the transcription of one gene suppresses the expression of its neighboring, oppositely transcribed counterpart. Excludons have been identified across evolutionarily distant bacterial species, suggesting that this is a widespread and conserved regulatory mechanism. Thus, they have been implicated in flagellum assembly in *Listeria monocytogenes* (Toledo-Arana et al., 2009), nitrogen metabolism in cyanobacteria (Álvarez-Escribano et al., 2024), energy production in *Edwarsiella piscicida* (Lanza et al., 2024), antibiotic adaptation (Sanmartín et al., 2025), and the balance between immunity and autoimmunity in *Staphylococcus aureus* phage biology (Rostol et al., 2024). Here, we used a web-based tool to generate an *H. influenzae* excludon map based on transcriptomic data (Sanmartín et al., 2025). As seen in other species, *H. influenzae* harbors more convergent (overlapping at 3´-UTRs) than divergent excludons (overlapping at 5´-UTRs). Among the identified convergent excludons, one particularly relevant example involves the *mtr* and *sdaCA* genes. This is noteworthy because it reveals a post-transcriptional regulatory connection between tryptophan and serine metabolism where antisense repression on *mtr* is exerted by the *sdaCA* 3´-UTR-overlapping mRNA. Serine is a direct precursor in one branch of the tryptophan biosynthetic pathway, where it combines with indole via the TrpA/TrpB enzyme complex to produce tryptophan. Expression of *mtr* increases in the presence of exogenous serine, whereas the expression of the *sdaCA* operon decreases, suggesting that the expression of both neighbor convergent genes is coordinated through 3′-UTR-overlapping transcription. These findings highlight the relevance of integrating excludon mapping into comparative transcriptomics and specifically add another layer to the functional and regulatory integration between tryptophan and serine metabolism in *H. influenzae*.

Moreover, we made several convergent observations regarding tryptophan transport as (i) expression of the *mtr* gene decreases in the presence of exogenous tryptophan; (ii) *tnaB* gene expression is lower than that of *mtr* in CDM-2, but this trend goes opposite when the medium is supplemented with tryptophan; (iii) same thing happens when the *mtr* gene is inactivated, as expression of the *tnaB* gene increases; (iv) expression of *mtr* is downregulated by the *sdaCA* 3′-UTR-overlapping transcription. Overall, although when either tryptophan transporter is inactivated the expression of the other increases, suggesting possible adaptation to tryptophan availability, data support a predominant role for TnaB in tryptophan uptake, not compensated by increased *mtr* gene expression upon *tnaB* inactivation. A NTHi375Δ*mtr*Δ*tnaB* double mutant may contribute explaining the benefits of exogenous tryptophan to *H. influenzae* survival. We acknowledge that, despite multiple trials, we were unable to generate this strain, leaving this aspect open for future work.

From the host perspective, the observed up-regulation of *ido-1* expression alongside down-regulation of genes involved in AhR signaling during *H. influenzae* infection under standard dietary tryptophan conditions seems counterintuitive, as IDO activity increases the availability of AhR ligands. Infection seems to overcome such IDO positive effect by lowering expression of the *ahr* gene, even in the presence of the AhR ligand FICZ. The observed AhR signaling suppression may facilitate enhanced pro-inflammatory responses (Euba et al., 2023), as observed for the *kc*, *tnf-α*, *il-6* and *il-1β* pro-inflammatory genes. Up-regulation of *ido-1* expression in response to lung infection by *M. tuberculosis* and *Francisella tularensis* has also been reported (Peng and Monack, 2010; Gautam et al., 2018), further supporting that tryptophan biosynthesis is an adaptation for pathogen survival within the host during tryptophan catabolism by IDO. Likewise, decreased body weight without affecting food intake has been previously reported in mice administered a tryptophan-free diet which may relate, among others, to lower body fat and low free-tryptophan content in muscle resulting from the tryptophan-depleted diet (Yokogoshi and Ashida, 1975; Kantak et al., 1980; van Winkle et al., 1983; Isales et al., 2020).

Moving forward the notion of bacterial metabolism as a source of therapeutics (Nogales and Garmendia, 2022), bacterial metabolic end products can also act as natural antibiotics, as it is the case for the TrpE allosteric inhibitor IPA. IPA *in vitro* inhibitory effects on *M. tuberculosis* (Negatu et al., 2018; Negatu et al., 2019) and resemblance between our modelled IPA-TrpE_NTHi375_ interactions and the previously shown tryptophan-TrpE interactions in *S. marcescens* (Spraggon et al., 2001), led us to hypothesize and confirm that IPA inhibits NTHi growth in a dose-dependent and reversible manner. Despite the clear *in vitro* activity, previous studies reported a lack of *in vivo* efficacy of IPA on *M. tuberculosis* lung infection (Negatu et al., 2018), suggesting that IPA alone may not be an ideal standalone therapy for pulmonary infections. Here, same observations were made on *H. influenzae* lung infection, together suggesting that future studies should explore developing antimicrobial regimes that incorporate tryptophan biosynthesis inhibition as a complementary strategy.

Overall, we highlight the critical importance of bacterial intracellular tryptophan pool for *H. influenzae* survival, as disruption of tryptophan biosynthetic genes impairs bacterial fitness *in vivo*, and tryptophan availability does not seem to influence pathogen burden in the lungs while the bacterial biosynthetic capability is maintained. These findings, next to novel evidence for a multifaceted fine-tuned regulation of exogenous tryptophan and serine uptake, highlight the potential of targeting *H. influenzae* tryptophan biosynthetic pathway as a therapeutic avenue, as suggested by the IPA *in vitro* inhibitory effects observed in this study.

## Materials and methods

### Bacterial strains and growth conditions

Strains used in this study are listed in **Supplementary Table 1**. *H. influenzae* strains were grown at 37 °C, 5% CO_2_ on PolyViteX agar (PVX, bioMérieux, 43101) or on *Haemophilus* Test Medium agar (HTM, Oxoid, CM0898), supplemented with 10 μg/mL hemin (Merck, H9039) and 10 μg/mL β-nicotinamide adenine dinucleotide (β-NAD, Merck, N0632), referred to as sHTM agar. *H. influenzae* liquid cultures were grown at 37 °C, 5% CO_2_ in chemically defined medium (CDM-2) (Gil-Campillo et al., 2025b). When indicated, CDM-2 was supplemented with L-tryptophan 0.5 mM (Merck, T0254), L-serine 10 mM (Merck, S4500), or indole 0.5 mM (Merck, I3408). Stock solutions were prepared as it follows: tryptophan 50 mM, dissolved in distilled water; serine 0.47 M, dissolved in distilled water; indole 1 M, dissolved in dimethyl sulfoxide (DMSO). When needed, *H. influenzae* liquid cultures were grown in brain heart infusion (BHI, Condalab, 1400.10) supplemented with hemin and β-NAD, referred to as sBHI. Erythromycin 11 μg/mL (Erm_11_) or Spectinomycin 50 μg/mL (Spec_50_) were used when required. *Escherichia coli* was grown on Luria Bertani (LB) or LB agar at 37 °C, with Ampicillin 100 μg/mL (Amp_100_), Erm_150_ or Spec_50_, when necessary.

*H. influenzae* strains were grown on PVX agar for 12 h. Depending on the assay, growth was next monitored as it follows: (i) two to five colonies were inoculated in 10 mL CDM-2, in the absence or presence of tryptophan or serine, and incubated for 12 h with shaking (100 r.p.m.). Cultures were then diluted to OD_600_ = 0.07 in the same medium and incubated in 25 mL CDM-2 in 250 mL flasks with shaking (200 r.p.m.), while OD_600_ was recorded every hour for up to 8 h. Experiments were performed on at least three independent occasions (*n* ≥ 3) (data shown in **Figure 1**). (ii) Bacterial biomass was collected from PVX agar, suspensions were normalized to OD_600_ = 0.4 in CDM-2, in the absence or presence of tryptophan or indole, and 100 µL aliquots were transferred to individual wells in 96-well flat bottom plates (Sarstedt, 82.1581.001) where 100 µL CDM-2 (with or without tryptophan or indole supplementation)/well had been previously added (final volume, 200 µL/well). Plates were incubated at 37 °C for up to 10 h in a Spectro Star Nano (BGM Labtech), and OD_600_ was measured in 15 min intervals. Each growth curve was corrected to its respective blank values (CDM-2). Experiments were performed in triplicate on at least three independent occasions (*n* ≥ 3) (data shown in **Figures 3** to **5**). (iii) *H. influenzae* strains were grown on sHTM agar with Erm_11_ for 16 h. Then, two to five colonies were inoculated in 10 mL CDM-2 with Erm_11_, in the absence or presence of tryptophan, and incubated for 12 h with shaking (100 r.p.m). Cultures were then diluted to OD_600_ = 0.07 in 15 mL of CDM-2 with Erm_11_, in the absence or presence of tryptophan, and incubated in 50 mL flasks with shaking (200 r.p.m.). After 8 h, 200 µL aliquots of stationary phase grown cultures (OD_600_ = 1.3) were transferred to 96-well plates (Nunc Optical Bottom plates with opaque polystyrene, Fisher Scientific, 165305) for fluorescence quantification in a SynergyH1 (BioteK) microplate reader. Fluorescence signal was quantified using a monochromator-based setting with specific excitation and emission wavelengths: green fluorescence protein (GFP) at 485/515 nm; red fluorescence protein (mCherry) at 587/645 nm. Each fluorescence signal was corrected to its respective blank values (CDM-2). Experiments were performed in triplicate on at least three occasions (*n* ≥ 3) (data shown in **Figure 2**).

### Determination of metabolite concentrations

Bacterial and mouse lung samples were used for metabolite quantification. For bacterial sample preparation, NTHi375 was grown for 12 h on PVX agar and two to five colonies were inoculated into 10 mL CDM-2 in the absence or presence of tryptophan or serine, grown for 12 h at 100 r.p.m., diluted into 20 mL fresh CDM-2 (same conditions), and grown for 8 h. At the indicated time points, 1 mL sample cultures were pelleted (5 min, 4 °C, 14–000 r.p.m.) and supernatants were transferred into new tubes; both pellets and supernatants were stored at -20 °C until use.

For metabolite determination two procedures were employed. *(i) Enzymatic measures.* Acetate concentrations were determined using the K-ACETRM kit, purchased from Megazyme, according to the manufacturer instructions. Acetate was not detected in CDM-2 medium control samples (data not shown). *(ii*) *High performance liquid chromatography (HPLC).* Bacterial intracellular tryptophan was extracted from bacterial pellets following a previously described method (Kim et al., 2016) with minor modifications. Briefly, bacterial pellets were washed with cold (4 °C) PBS twice, 400 μL ice-cold 50% methanol/50% HEPES-EDTA and 400 μL ice-cold chloroform were added, vigorously vortexed for 45 min at -20 °C, and centrifuged at 14.000 r.p.m. at 4 °C for 10 min. Then, the upper water/methanol phase, containing the extracted metabolites, was collected and frozen at -20 °C. Chromatographic separation for tryptophan quantification was performed using a Waters Alliance HPLC system (Waters, Milford, MA, USA) connected to a fluorescence detector and a AccQ Tag column (150 x 3.9 mm i.d., particle size = 4 μm; Waters) with an isocratic mobile phase of 15 mM potassium phosphate (pH 6.4), with 2.7% (v/v) acetonitrile with a flow of 0.8 mL/min and 30 °C column temperature. The fluorescence detector was operated at 280 nm excitation and 340 nm emission wavelengths. For indole quantification the same HPLC system described above connected to a PDA detector and to a Luna Omega C18 100 Å column (250 x 4.6 mm i.d., particle size = 5 μm; Phenomenex), was used. A gradient elution with H_2_O-0.1% (v/v) formic acid and acetonitrile as the mobile phases at a flow rate of 1 mL/min (65:35 for 0–5 min, 35:65 for 5–12 min, and 65:35 at 12 min) and 25 °C column temperature were set. Indole was monitored at 271 nm. For amino acid determination, supernatant samples were derivatized using the AccQ-Tag method (Waters Associates, Milford, Mass., USA). To separate the amino acids, the same HPLC system described above equipped with a reversed-phase column (AccQ Tag column, 150 x 3.9 mm i.d., particle size = 4 μm, Waters) and a fluorescence detector (excitation wavelength 280 nm; emission wavelength 340 nm) was used. The gradient was accomplished with buffer A containing 140 mM sodium acetate (pH 5.8) and 7 mM triethanolamine. Acetonitrile and water were used as eluents B and C. The flow rate was set at 1 mL/min, and the column was heated at 37 °C during the whole measurement. The gradient was produced by the following concentration changes: 1 min 1% B, 27 min 5% B, 28.5 min 9% B, 44.5 min 18% B, 47.5 min 60% B and 40% C, hold for 3 min and return to 0% B in 1 min. Glucose content in supernatants was measured by HPLC with pulsed amperometric detection using a DX-3000 Dionex system (Dionex, Sunnyvale, CA). Separation was carried out on a CarboPac PA20 column (150 x 3 mm i.d.; Thermo-Scientific) at 30 °C with an isocratic elution in 15% 0.3 M NaOH.

Frozen stored lungs were used for tryptophan determination. Same metabolite extraction method as for bacterial intracellular tryptophan was followed (see above); lungs were previously homogenized in 400 μL ice-cold 50% methanol/50% HEPES-EDTA using Ultra-Turrax (IKA, 3951600). Tryptophan concentration was determined using the chromatographic methods described above for intracellular bacterial tryptophan determinations. Tryptophan, indole, amino acids and glucose were quantified based on standard calibration curves generated with commercial compounds.

### Bacterial RNA extraction, purification, sequencing and data analysis

NTHi375 was grown for 12 h on PVX agar. Two to five colonies were inoculated into 10 mL CDM-2, in the absence or presence of tryptophan or serine, grown for 12 h at 100 r.p.m., diluted into 20 mL fresh CDM-2 to OD_600_ = 0.07, and grown to OD_600_ = 0.6 at 200 r.p.m. Next, 7 mL bacterial cultures were recovered, pelleted (4,000 r.p.m., 4 min), flash frozen, and stored at -80 °C. Bacterial RNA was isolated using NucleoSpin RNA kit (Macherey-Nagel) as specified by the manufacturer´s instructions. Briefly, ∼5x10^9^ CFU (3 mL cultures) were pelleted by centrifugation at 14,000 r.p.m. for 5 min, resuspended in 100 µL TE buffer (10 mM Tris-HCl, 1 mM EDTA, pH 8) containing 1 mg/mL lysozyme by vigorous vortexing, and incubated at 37 °C for 10 min. Cells were lysed with 350 μL Buffer RA1 and 3.5 μL β-mercaptoethanol. Lysates were filtered through NucleoSpin Filter units to reduce viscosity and mixed with 350 μL 70% ethanol. RNA was applied to NucleoSpin RNA columns, salt was removed using membrane desalting buffer (MDB), one on-column rDNase treatment step was included, samples were cleaned with RAW2 and RA3 buffers, and RNA was eluted. RNA quality was evaluated using RNA 6000 Nano LabChips (Agilent 2100 Bioanalyzer, Santa Clara, CA) and sequenced on an Illumina HiSeq platform with 2 × 150 bp reads by Admera Health. Sixty million 2 × 150 bp reads were generated for the three replicates of each sample type. RNAseq raw sequencing data reads were deposited in the NCBI Sequence Read Archive (SRA) and are available under BioProject number PRJNA1158978.

From the RNA-seq reads, the adapters were removed from the sequenced libraries using TrimGalore v0.6.10 (www.bioinformatics.babraham.ac.uk/projects/trim_galore/) with default settings for paired-end Illumina reads. Reads shorter than 20 bp or with an error rate (TrimGalore option “-e”) higher than 0.1 were discarded. Prior to the mapping step, the genome was indexed using STAR v2.7.10b (Dobin et al., 2013) with the parameters –runMode genomeGenerate and –genomeSAindexNbases 9.4. The remaining reads were then mapped to the NTHi375 genome (GCA_000012185.1) using STAR with default parameters, except for enabling “–twopassMode basic.” Duplicated reads were marked prior to quantification using Picard tools v3.0 (https://broadinstitute.github.io/picard/). Quantification was performed using HTSeq-count, as part of HTSeq v.2.0.5 (Putri et al., 2022), with the NCBI annotation in GTF format and the option “–stranded no.” Statistical analysis and FPKM calculations were conducted with the DESeq2 v1.44.0 (Love et al., 2014) package in R. Expression tracks were generated using the bamCoverage function in deepTools v3.5.0 (Ramírez et al., 2016), with the parameter –normalize Using RPKM. Mean RPKM values of differentially regulated genes from each sample in each experimental group were used to perform PCA analysis using ClustVis web tool1 with default parameters. NTHi375 differentially expressed genes, pathways or biological functions, with fold change >1.5, were searched in KEGG, Pubmed and Uniprot; BLASTn was performed using the sequence of each differentially expressed bacterial gene as query against all *H. influenzae* genomes available at NCBI. Remaining unidentified genes were grouped as hypothetical.

Transcriptional maps were generated by converting the obtained read coverage files (.wig) from the plus and minus strands to BigWig (.bw) files using the wig2BigWig program. The generated.bw files and the gene annotation files were loaded into a local web server based on Jbrowse for visualization (Skinner et al., 2009).

### Excludon map of *H. influenzae*

To systematically identify the excludons in the *H. influenzae* genome, we employed the ExcludonFinder computational tool (version 1.0, August 27th 2025) (https://excludonfinder-unavarra.com) using the web-based interface (Sanmartín et al., 2025). Subsequent versions of the tool incorporate improvements implemented based on user feedback and suggestions from the scientific community following its initial release. Overall results are expected to remain practically identical, although minor differences in the output may occur. RNA-seq data from the CDM-2 control condition in BioProject PRJNA1158978 were analyzed using the *H. influenzae* NTHi375 reference genome (CP009610.1) and its corresponding annotation file. The analysis was performed with default parameters, setting the coverage threshold to 0.5 (50%) to define transcriptional boundaries.

### Generation of transcriptional reporter plasmids

Plasmids and primers used in this study are listed in **Supplementary Table 1** and **Supplementary Table 2**, respectively. The pTBH03-Prom-less plasmid (Rapún-Araiz et al., 2023) was used to amplify a 800 bp fragment containing the *gfp* gene and *mtr* promoter region (the later included in primer 2297), using primers 2296 and 2297. This Pr*_mtr_::gfp* amplicon was cloned into pJET1.2/blunt (pJET1.2-Pr*_mtr_::gfp*), digested with *Sph*I and *Asc*I, and cloned into pTBH03-Prom-less previously digested with *Sph*I and *Asc*I, to generate pTBH03-Pr*_mtr_::gfp*. A 200 bp fragment containing the *sdaCA* promoter region was amplified using NTHi375 genomic DNA as template with primers 2294 and 2295, cloned into pJET1.2/blunt (pJET1.2-Pr*_sdaCA_*), digested with *Sph*I and *Spe*I, and cloned into pTBH03-Prom-less previously digested with *Sph*I and *Spe*I to generate pTBH03-Pr*_sdaCA_::gfp*. A commercially synthesized (GenScript) 1,900 bp DNA fragment containing the Pr*_sdaCA_::gfp-*Pr*_mtr_::m-cherry* sequence was excised from a pUC18 derivative plasmid (pUC18-*Pr_sdaCA_::gfp-Pr_mtr_::m-cherry*) by *Sph*I and *Asc*I digestion, and ligated into the *Sph*I and *Asc*I digested pTBH01 plasmid (Fernández-Calvet et al., 2021), generating pTBH-*Pr_sdaCA_::gfp-Pr_mtr_::m-cherry*, named pSGMC. pTBH03-Prom-less (Rapún-Araiz et al., 2023) was used to amplify a 800 bp fragment containing the *gfp* gene and transcription terminator from pSEVA (included in the 2468 primer sequence) using primers 2400 and 2468. This amplicon was cloned into pJET1.2/blunt (pJET1.2-GFP.TT), and then excised by *Sph*I and *Bam*HI digestion. pUC18-*Pr_sdaCA_::gfp-Pr_mtr_::m-cherry* was digested with *Asc*I and *BamH*I to excise the *Pr_mtr_::m-cherry* containing fragment. Both *Pr_mtr_::m-cherry* and *gfp* gene-transcription terminator fragments were cloned into *Sph*I and *Asc*I digested pTBH01 generating pTBH-Δ*Pr_sdaCA_::gfp-Pr_mtr_::m-cherry*, named pGMC. These plasmids were independently electroporated into *H. influenzae* RdKW20 (Fleischmann et al., 1995). Transformants were selected on sHTM agar with Erm_11_.

### *sdaCA-mtr* region transcript overlapping detection by RT-PCR

RdKW20 (pSGMC) and RdKW20 (pGMC) were used for RNA extraction (see below). Next, 2 µg of RNA per sample were retrotranscribed using M-MLV retrotranscriptase (Promega, M1701) and 10 mM of the 3´ primer 2610 designed for analyzing *sdaCA*/GFP gene, following manufacturer´s instructions. After retrotranscription, 1 µL cDNA was used as a template for a 30-cycle PCR reaction using DreamTaq DNA polymerase (Fisher Scientific, 15699364) with a primer pair designed to analyze the *sdaCA* locus, primers 2610 and 2609. As a negative control, a PCR reaction was performed with 1 µL of non-reversely transcribed RNA. As a control of primers performance, a PCR reaction was performed using pTBH-derived plasmids DNA.

### Bacterial and host RNA extraction, purification and RT-qPCR analyses

RNA was isolated from two sample types: *(i) Bacterial cultures.* NTHi375 and RdKW20 plasmid-containing derivative strains were grown for 12 h on PVX agar or sHTM agar with Erm_11_. Two to five colonies were inoculated into 10 mL CDM-2, in the absence or presence of tryptophan, with Erm_11_ when needed, grown for 12 h at 100 r.p.m., diluted into 20 mL fresh CDM-2 (same conditions) to OD_600_ = 0.07, and grown at 200 r.p.m. to OD_600_ = 0.6 or 1.2. In all cases, 7 mL were pelleted (4,000 r.p.m., 10 min), flash frozen, and stored at -80 °C. Total RNA was isolated using TRIzol reagent (Invitrogen, 15596026). *(ii) Mouse lung samples*. Mouse lungs were thawed (see below), homogenized in 1 mL TRIzol using Ultra-Turrax (IKA, 3951600), and total RNA was isolated using TRIzol reagent.

In all cases, reverse transcription was performed using 1 μg RNA by PrimerScript RT Reagent kit (Takara). cDNA was 1:10 diluted and was used as template for qPCR. In all cases, 20 μL reaction mixtures containing 1X SYBR Premix Ex Taq II (Tli RNaseH Plus) (Takara) and adequate primer pairs, designed with Primer3 or Vector NTI (Life Technologies) software, were used. Fluorescence data were analyzed with AriaMx Real-Time PCR System (Agilent Technologies) and QuantStudio 5 Real-Time PCR system (Thermofisher). The comparative threshold cycle (Ct) method was used to obtain relative quantities of mRNA that were normalized using bacteria *gyrA* gene, or mouse *gapdh* as endogenous controls (values ± SD). All measures were performed in triplicate and at least three times (n≥3).

### Generation of *H. influenzae* mutant strains

For *trpB* gene inactivation, a 2,530 bp DNA fragment corresponding to the *trpB* ORF (1,194 bp) and 607 bp upstream and 730 bp downstream flanking regions, was PCR amplified using NTHi375 genomic DNA as template with primers TrpB-F1 (2258) and TrpB-R1 (2259), and cloned into pJET1.2/blunt (Fisher Scientific), generating pJET1.2-*trpB* (P1293). pJET1.2-*trpB* was linearized by inverse PCR using primers TrpB-F2 (2260) and TrpB-R2 (2261) to disrupt the *trpB* gene, followed by ligation to a blunt-ended Erm resistance cassette obtained from pBSLerm *Sma*I digestion (Allen et al., 2005) to generate pJET1.2-*trpB*::*ermC* (P1294). The *trpB::ermC* disruption cassette (2,750 bp) was next PCR amplified with primers TrpB-F1 (2258) and TrpB-R1 (2259). This amplicon was used for NTHi375 natural transformation using the M-IV method (Herriott et al., 1970). NTHi375Δ*trpB::ermC* (P1295) mutant strain was selected on sHTM agar with Erm_11_. For *trpDCF* gene inactivation, a 4,000 bp DNA fragment corresponding to the *trpDCF* ORF (2,457 bp) and 653 bp upstream and 810 bp downstream flanking regions, was PCR amplified using NTHi375 genomic DNA as template with primers TrpDCF-F1 (2262) and R1-trpDCF (2254), and cloned into pJET1.2/blunt to generate pJET1.2-*trpDCF* (P1298). pJET1.2-*trpDCF* was linearized by inverse PCR using primers TrpDCF-F2 (2263) and R2-trpDCF (2056), followed by ligation to a blunt-ended Erm resistance cassette obtained from pBSLerm *Sma*I digestion to generate pJET1.2-*trpDCF*::*ermC* (P1299). The *trpDCF::ermC* disruption cassette (2,880 bp) was PCR amplified with primers TrpDCF-F1 (2262) and R1-trpDCF (2054), and this amplicon was used for NTHi375 natural transformation with the M-IV method. NTHi375Δ*trpDCF::ermC* (P1193) mutant strain was selected on sHTM agar with Erm_11_. To disrupt the *mtr* gene, a 3,098 bp DNA fragment corresponding to the *mtr* ORF (1,257 bp), and 979 bp upstream and 862 bp downstream flanking regions, was PCR amplified using NTHi375 genomic DNA as template with primers mtr-F1 (2057) and mtr-R1 (2058), and cloned into pJET1.2/blunt to generate pJET1.2-*mtr* (P1091). pJET1.2-*mtr* was linearized by inverse PCR using primers mtr-F2 (2080) and mtr-R2 (2081), followed by ligation to a blunt-ended Spec resistance cassette obtained from pRSM2832 by *EcoR*V digestion (Tracy et al., 2008) to generate pJET1.2-*mtr*::*spec* (P1092). The *mtr::spec* disruption cassette (3,961 bp) was PCR amplified with primers mtr-F1 (2057) and mtr-R1 (2058), and this amplicon was used for NTHi375 natural transformation using the M-IV method. NTHi375Δ*mtr::spec* (P1194) mutant strain was selected on sHTM agar with Spec_50_. To disrupt the *tnaB* gene, a 1,900 bp DNA fragment corresponding to the *tnaB* ORF (1,107 bp) and 703 bp downstream region was PCR amplified using NTHi375 genomic DNA as template with primers *tnaB*-F2 (2187) and *tnaB*-R1-NTHi375 (2163), and cloned into pJET1.2/blunt to generate pJET1.2-*tnaB* (P1201). pJET1.2-*tnaB* was linearized by inverse PCR using primers *tnaAB*-F2-NTHi375 (2150) and *tnaA*-R1-NTHi375 (2147), followed by ligation to a blunt-ended Spec resistance cassette obtained from pRSM2832 *EcoR*V digestion to generate pJET1.2-*tnaB*::*spec* (P1202). The *tnaB::spec* disruption cassette (2,610 bp) was PCR amplified with primers *tnaB*-F2 (2187) and *tnaB*-R1-NTHi375 (2163) from pJET1.2-*tnaB*::*spec*, and this amplicon was used for NTHi375 natural transformation with the M-IV method. NTHi375Δ*tnaB::spec* (P1203) mutant strain was selected on sHTM agar with Spec_50_. In all cases, mutants were confirmed by PCR.

### Protein structure and tryptophan docking prediction

The structure of TrpE_NTHi375_ was predicted using AlphaFold2 (AF2) (Jumper et al., 2021) with a high degree of confidence. The predicted structure exhibits significant similarity to other structures available in the Protein Data Bank (PDB), such as *S. marcescens* (PDB ID 17IS). NTHi375 AF2 model was prepared and refined using the Protein Preparation Workflow in Maestro (Schrödinger Release 2022-2). The LigPrep module was employed to prepare and refine the IPA ligand molecule to generate several potential conformations. Glide (Friesner et al., 2004) was subsequently used to dock all ligand conformers in extra-precision (XP) mode. A grid box of dimensions 25 Å × 25 Å × 25 was defined to encompass the active site of TrpE_NTHi375_. The best-scoring ligand pose was selected and further minimized. Throughout all procedures, the OPLS4 force field was applied, with default settings retained.

### Determination of IPA antimicrobial effects

Indole propionic acid (IPA) (Merck, 220027) was used by freshly preparing a 0.4 M stock solution in DMSO. IPA susceptibility was determined as follows: the 0.4 M stock solution was diluted to 0.6, 1.2, 1.8 and 2.4 mM working solutions in CDM-2. Next, 100 µL aliquots of each IPA working solution were transferred to individual wells in 96-well flat bottom plates. A suspension of PVX agar freshly grown bacteria was generated in CDM-2, adjusted to OD_600_ = 0.4. Then, 100 µL bacterial aliquots were transferred to each well. Plates were incubated at 37 °C for up to 24 h in a Spectro Star Nano (BGM Labtech), and OD_600_ was measured in 15 min intervals.

To assess IPA effect reversibility, assays were set as indicated above. After 24 h of incubation, cultures were serially 10-fold diluted in PBS, plated in triplicate on sHTM agar to determine the number of viable bacteria, and passaged by adding 20 µL bacterial aliquots into 180 µL fresh CDM-2, to individual wells in 96-well flat bottom plates. Plates were incubated for 24 additional h and OD_600_ was measured as above. Then, cultures were serially 10-fold diluted in PBS and plated in triplicate on sHTM agar.

To assess possible IPA effect restoration, a 0.4 M IPA stock solution was diluted to 1.2 and 1.8 mM working solutions in CDM-2, supplemented with tryptophan or indole, and 100 µL aliquots of each IPA working solution were transferred to individual wells in 96-well flat bottom plates. Bacterial suspensions were prepared as indicated above, and 100 µL bacterial aliquots were transferred to each well. Plates were incubated at 37 °C for up to 24 h in a Spectro Star Nano (BGM Labtech), and OD_600_ was measured in 15 min intervals. Bacterial growth controls (without IPA and with DMSO) were included in all cases. All assays were performed in triplicate at least three times (n ≥ 3).

### Animal experiment zprocedures

CD1 female mice (18–20 g) aged 4 to 5 weeks (Charles River Laboratories) were housed under pathogen-free conditions at the IdAB-CSIC animal facility (registration number ES/31-2016-000002-CR-SU-US). Animal handling and procedures were in accordance with European (Directive 2010/63/EU) and National (RD118/2021) legislation, with authorization of the CSIC Ethics Committees, and local Government (Protocol PI007/19). When indicated, porcine pancreatic elastase (PPE, Elastin Products Company) was administered in mouse previously anesthetized with isoflurane (Zoetis) for emphysema induction, with matching vehicle solution control groups as described (Rodríguez-Arce et al., 2021). When indicated, either IPA or vehicle solution were administered by oroesophageal gavage (Popper&Sons Inc.). Unless indicated, mice were administered a standard diet; when specified, animals were administered a tryptophan-depleted diet (Inotiv, MD.08126-Tryptophan Deficient HIIRAT.8) 21 days before infection. When indicated in animals receiving a tryptophan-depleted diet, 6-formylindolo[3,2-b]carbazole (FICZ) (Merck, SML1489) or vehicle solution were administered by intraperitoneal injection.

To prepare bacterial infecting inocula, NTHi375 derivative strains were grown on PVX agar for 12 h; next, 2 to 5 colonies were inoculated in 10 mL CDM-2 (tryptophan addition indicated when needed), and incubated for 11 h with shaking (100 r.p.m.). Cultures were diluted to OD_600_ = 0.07 in 25 mL CDM-2 (same as above) and grown in triplicate in 250 mL flasks with shaking (200 r.p.m.) up to OD_600_ = 0.3, when 1 mL cultures were stored at -80°C. When needed, NTHi375 derivative strains were grown on PVX agar for 12 h; next, 2 to 5 colonies were inoculated in 10 mL sBHI, and incubated for 11 h with shaking (100 r.p.m.). Cultures were diluted to OD_600_ = 0.07 in 25 mL sBHI and grown in triplicate in 250 mL flasks with shaking (200 r.p.m.) up to OD_600_ = 0.3, when 1 mL cultures were stored at -80°C.

In all cases, mice were intranasally infected by placing a 20 μL bacterial suspension containing ~1x10^8^ CFU/mouse at the entrance of the nostrils until complete inhalation by each mouse, previously anesthetized with ketamine (Imalgene^®^, Merial) and xylazine (Rompun^®^, Bayer AG) (3:1). At 24 h post-infection (hpi), mice were euthanized by cervical dislocation. BALF samples were obtained by perfusion and collection of 0.7 ml of PBS, with help of a sterile 20G (1.1 mm diameter) VialonTM intravenous catheter (Becton-Dickinson) inserted into the trachea. Recovered BALF samples were serially 10-fold diluted in PBS and plated in triplicate on sHTM agar to determine the number of viable bacteria. By following standardized published procedures, we considered that we could have a minimum of 3.3 c.f.u. in 1 mL of sample without detecting bacteria (limit of detection < 3–4 c.f.u./mL BALF), rendering log_10_ = 0.52. Left lungs were aseptically removed, weighed in sterile bags (Stomacher80, Seward Medical) and homogenized 1:10 (w/v) in PBS. Each homogenate was serially 10-fold diluted in PBS and plated in triplicate on sHTM agar (in the absence or presence of antibiotic, see below), to determine the number of viable bacteria (CFU counts). When needed, the right lung was frozen stored for RNA extraction or tryptophan determination (see above).

Three types of assays were performed: *(i) Analysis of the effect of tryptophan and AhR signalling on H. influenzae infection*. To do so, 48 CD1 animals were divided into two groups: mice administered a standard diet (n=16); animals administered a tryptophan-depleted diet (n=32). When indicated, 6-formylindolo[3,2-b]carbazole (FICZ) 1 μg/mouse or vehicle solution (DMSO in PBS 1:15) were administered by intraperitoneal injection 1 h before infection. Animals were non-infected, or infected as above indicated and euthanized at 24 hpi (n≥6 per group). In infected groups, BALF and left lungs were processed for CFU counts as indicated above; in all non-infected and infected groups, right lungs were frozen stored for subsequent RNA extraction as indicated above.

*(ii) NTHi infection for competitive index (CI) determination.* Co-infections with wild-type (WT) and mutant (ratio 1:1) were performed (Gil-Campillo et al., 2025a). For this purpose, 42 CD1 mice with lung emphysema were used. WT and mutant strain suspensions were prepared and adequately mixed to prepare bacterial mix suspensions (1:1) containing 5x10^9^ CFU/mL. Mice were administered 20 μL (~1x10^8^ CFU/mouse, 5x10^7^ CFU/strain/mouse) by the intranasal route and sampled at 24 hpi (n=7 animals/mutant to be tested). Lungs were aseptically removed and processed as indicated above, in the absence or presence of antibiotic (Erm or Spec depending on strain, **Supplementary Table 1**). CFU counts were used for CI determination as (CFU_mutant_/CFU_WT_)_output_/(CFU_mutant_/CFU_WT_)_input_.

*(iii) Assessement of IPA effect on H. influenzae lung infection*. To do so, 64 CD1 animals were divided into two groups. The first group of mice (n=48) was treated with oral IPA at doses of 50 or 100 mg/kg of body weight, administered in 0.1 mL PBS-DMSO (1:3). Administrations were performed daily during 4 days before infection. Animals were infected as above indicated and euthanized at 24 or 30 hpi (n≥6 per group). A second group of mice (n=16) was administered oral IPA at a dose of 150 mg/kg of body weight in 0.1 mL PBS-DMSO (1:1). Administrations were performed daily during 3 days before infection and at 1 day post-infection. In all cases, control groups receiving vehicle solution (DMSO in PBS 1:3 or 1:1) were included in parallel. NTHi375 was used for infection as indicated above. Animals were euthanized at 30 hpi (n≥6 per group). In both cases, BALF and left lungs were processed for CFU counts as indicated above.

### Statistical analyses

In all cases, P<0.05 value was considered statistically significant. Analyses were performed using GraphPad Prism software version 7 for MacOS and are detailed in each Figure Legend.

## Data Availability

The data presented in the study are deposited in the NCBI repository, accession number PRJNA1158978.
