# Supplementary material for: Haemophilus influenzae tryptophan biosynthesis is required for lung infection
Source: Front Cell Infect Microbiol. 2026 Jul 10;16:1787089. doi: 10.3389/fcimb.2026.1787089 (PMC13395689; doi:10.3389/fcimb.2026.1787089)
Supplement: Supplementary file 5 [file Supplementaryfile1.pdf]

Haemophilus influenzae tryptophan biosynthesis is required for lung infection

Javier Asensio-López[1,2,3], Beatriz Rapún-Araiz[1,3], Begoña Euba[1,3], Asier Domínguez-San Pedro[1], Álvaro Sanmartín[4], Celia Gil-Campillo[1,3], David San León[5,6], Pablo Chacón[7], Goizeder Almagro[1], Ana Ardá[8], Saioa Burgui[2], Iñigo Lasa[4], Alejandro Toledo-Arana[1], Junkal Garmendia[1,3,9#]

[1]Instituto de Agrobiotecnología, Consejo Superior de Investigaciones Científicas (IdAB-CSIC)-Gobierno de Navarra, Mutilva, 31192 Navarra, Spain;

[2]Asociación de la Industria Navarra (AIN)-Gobierno de Navarra, Cordovilla, Spain;

[3]Centro de Investigación Biomédica en Red de Enfermedades Respiratorias (CIBERES), Madrid, Spain;

[4]Laboratory of Microbial Pathogenesis, Navarrabiomed-Universidad Pública de Navarra (UPNA)-Complejo Hospitalario de Navarra (CHN), IdiSNA, Irunlarrea 3, Pamplona, 31008 Navarra, Spain;

[5]Department of Systems Biology, Centro Nacional de Biotecnología, CSIC, Madrid, Spain;

[6]Interdisciplinary Platform for Sustainable Plastics towards a Circular Economy-Spanish National Research Council (SusPlast-CSIC), Madrid, Spain;

[7]Department of Biological Physical Chemistry, Rocasolano Institute of Physical Chemistry, CSIC, 28006 Madrid, Spain;

[8]Center for Cooperative Research in Biosciences (CIC bioGUNE), Basque Research and Technology Alliance (BRTA); Ikerbasque, Basque Foundation for Science, Bilbao, 48009, Spain;

[9]Conexión Antimicrobial Resistance-AMR, CSIC, Madrid, Spain

Content:

Dataset S1. Distribution of the *tnaCAB* locus across *H. influenzae* strains with complete and publicly available genome sequences.

Dataset S2. DEG in NTHi375 strain grown in CDM-2, in the absence or presence of Trp or serine.

Dataset S3. Convergent and divergent excludons identified in *H. influenzae* using ExcludonFinder.

Dataset S4. Excludon pairs displaying antagonistic differential expression under CDM-2 serine supplementation.

Table S1. Bacterial strains and plasmids used in this study.

Table S2. Primers used in this study.

Figure S1. Effect of tryptophan supplementation on *H. influenzae* gene expression.

Figure S2. Amino acid consumption by NTHi375 during growth in CDM-2 in the absence/presence of tryptophan.

Figure S3. Definition of NTHi375 *mtr* and *sdaCA* promoter regions.

Figure S4. Prediction of $TrpE_{NTHi375}$ structure.

Figure S5. Effect of IPA oral administration on NTHi375 murine respiratory infection.

Table S1. Strains and plasmids used in this study.

Strain / Plasmid	Description	Source
**Strain**		
E. coli		
TOP10	Cloning strain. F- *mcrA* Δ(*mrr-hsd*RMS-*mcr*BC) Φ80*lac*ZΔM15 Δ*lac*X74 *rec*A1 *ara*D139 Δ(*ara,leu*)7697 *gal*U*gal*K*rps*L (Str^R) *end*A1 *nup*G	Thermofisher Scientific
H. influenzae		
NTHi375	Wild-type, otitis media clinical isolate	(1)
NTHi375Δ*trpB* / P1295	*trpB::ermC*, Erm^R	This study
NTHi375Δ*trpDCF* / P1193	*trpDCF::ermC*, Erm^R	This study
NTHi375Δ*mtr* / P1194	*mtr::spec*, Spec^R	This study
NTHi375Δ*tnaB* / P1203	*tnaB::spec*, Spec^R	This study
RdKW20 (pTBH01) / P1085	RdKW20 derivative, transformed with pTBH01, a pACYC177 derivative containing an Erm^R gene and a MCS. Amp^R, Erm^R	(2)
RdKW20/P189	Wild-type, Laboratory strain, capsule-deficient serotype d	(3)
RdKW20 (pSGMC) / P1391	RdKW20 derivative, transformed with pSGMC plasmid carrying the *gfpmut2* and *mCherry* genes expressed under P*sdaCA* and P*mtr*, respectively. Amp^R, Erm^R	This study
RdKW20 (pGMC) / P1411	RdKW20 derivative, transformed with pGMC plasmid carrying the *gfpmut2* gene without promoter and *mCherry* gene expressed under P*mtr*. Amp^R, Erm^R	This study
RdKW20 (pTBH03-Pr$_{sdaCA}$::*gfp*) / P1325	RdKW20 derivative, transformed with pTBH03-Pr$_{sdaCA}$::*gfp* carrying the *gfpmut2* gene expressed under the promoter of *sdaCA* from NTHi 375. Amp^R, Erm^R	This study
RdKW20 (pTBH03-Pr$_{mtr}$::*gfp*) / P1326	RdKW20 derivative, transformed with pTBH03-Pr$_{mtr}$::*gfp* plasmid carrying the *gfpmut2* gene expressed under promoter of *mtr* from NTHi 375. Amp^R, Erm^R	This study
**Plasmid (*E. coli* strain carrying plasmid)**		
pJET1.2/blunt	Cloning vector	Life Technologies
pBSLerm	Plasmid containing an Erm resistance cassette (Erm^R)	(4)
pRSM2832	pKD13 derivative carrying a cassette containing a Spec resistance gene flanked by FRT sites	(5)
pJET1.2-*trpB* (P1293)	pJET1.2 derivative containing a 2,530 bp DNA fragment carrying the *trpB* gene (1,194 bp) and its upstream (606 bp) and downstream (730 bp) flanking regions	This study
pJET1.2-*trpB*::*ermC* (P1294)	pJET1.2-*trpB* derivative containing a 2,750 bp DNA fragment carrying a *trpB::ermC* disruption cassette	This study
pJET1.2-*trpDCF* (P1298)	pJET1.2 derivative containing a 4,000 bp DNA fragment carrying the *trpDCF* gene (2,457 bp) and its upstream (653 bp) and downstream (890 bp) flanking regions	This study
pJET1.2-*trpDCF*::*ermC* (P1298)	pJET1.2-*trpDCF* derivative containing a 2,880 bp DNA fragment carrying a *trpDCF::ermC* disruption cassette	This study
pJET1.2-*mtr* (P1091)	pJET1.2 derivative containing a 3,098 bp DNA fragment carrying the *mtr* gene (1,257 bp) and its	This study

	upstream (979 bp) and downstream (862 bp) flanking regions	
pJET1.2-*mtr::spec* (P1092)	pJET1.2-*mtr* derivative containing a 3,961 bp DNA fragment carrying a *mtr::spec* disruption cassette	This study
pJET1.2-*tnaB* (P1201)	pJET1.2 derivative containing a 1,901 bp DNA fragment carrying the *tnaB* gene (1,107 bp) and downstream (794 bp) flanking region	This study
pJET1.2-*tnaB*::*spec* (P1202)	pJET1.2-*tnaB* derivative containing a 2,610 bp DNA fragment carrying a *tnaB::spec* disruption cassette	This study
pTBH03-Prom-less (P1177)	pTBH01 derivative, containing the *gfpmut*2 gene, without promoter. A fragment of 750 bp was digested with *EcoR*I and *Asc*I from pTBH03 and ligated into pTBH01 (Amp^R, Erm^R)	(6)
pTBH03 (P1123)	pTBH03-Pr_{hmw} derivative expressing *gfpmut*2 under the Phyper promoter, with Gram-negative RBS.	(6)
pJET1.2-Pr_{mtr}::*gfp* (P1327)	pJET1.2 derivative containing a 800 bp DNA fragment carrying the promoter region of the *mtr* gene and the fluorescence protein GFP	This study
pJET1.2-Pr_{sdaCA} (P1328)	pJET1.2 derivative containing a 200 bp DNA fragment carrying the *sdaCA* promoter region	This study
pTBH03-Pr_{mtr}::*gfp* (P1333)	pTBH03-Prom-less derivative containing a 800 bp DNA fragment carrying the promoter region of the *mtr* gene and the fluorescence protein GFP.	This study
pTBH03-Pr_{sdaCA}::*gfp* (P1332)	pTBH03-Prom-less derivative containing 200 bp DNA fragment carrying the *sdaCA* promoter region.	This study
pJET1.2-GFP.TT (P1368)	pJET1.2 derivative containing a 800 bp DNA fragment carrying the *gfpmut*2 gene and the upstream pSEVA transcription terminator without a promoter	This study
pUC18- Pr_{sdaCA}::*gfp*-Pr_{mtr}::*m-cherry*	pUC18 derivative carrying the *sdaCA* promoter with the *gfp gfpmut*2 gene and pSEVA terminators, and the *mtr* promoter with the *m-cherry* gene and the intergenic region between both *sdaCA* and *mtr*. This DNA fragment was obtained by synthesis (GeneScript)	This study
pSGMC (P1371)	pTBH01 derivative containing a 1900 bp DNA fragment carrying the *sdaCA* promoter with the *gfpmut*2 gene and pSEVA terminators, and the *mtr* promoter with the *m-cherry* gene and the intergenic region between both *sdaCA* and *mtr* reporters.	This study
pGMC (1372)	pTBH01 derivative containing a 1600 bp DNA fragment carrying carrying the *gfpmut2* gene without promoter and *mCherry* gene expressed under P*mtr* and the intergenic region between both *sdaCA* and *mtr*. Amp^R, Erm^R	This study

Table S2. Primers used in this study.

Primer name	PrimerID	Primer sequence (5'-3')	Purpose	Source
TrpB F1	2258	GATATTCGTGATGAACAAGTCACAATT	Disruption cassette	This study
TrpB R1	2259	CGCTCAATAATTTTTACTGTTGCCGAA	Disruption cassette	This study
TrpB F2	2260	AACACGAAGGCATTATTCCTGCATTAG	Disruption cassette	This study
TrpB R2	2261	CAAATTCTGCTTGGAATGTAGGATCAT	Disruption cassette	This study
TrpDCF F1	2262	ACAAGATTTACCAAAAGAATTTATCGT	Disruption cassette	This study
R1-*trpDCF*	2054	GAAGTGATCGTTGCGGTGCGTAATATT	Disruption cassette	This study
TrpDCF F2	2263	GTGAAACAAGCCATTCAACAAAATTGC	Disruption cassette	This study
R2-*trpDCF*	2056	GGAAAGCTCACCTTGGATGACTGCATT	Disruption cassette	This study
mtr-F1	2057	ATGGCGATGAAGTGCTTGTGCCGATGC	Disruption cassette	This study
mtr-R1	2058	TGTTAGTGCTCATCTAGAGAATGTCTG	Disruption cassette	This study
mtr-F2	2080	TCCTTGTGTACTACTATAATAGTGCAAA TTTATTTTAGAGGCATTTTATGATTCCGGGG ATCCGTCGACC	Disruption cassette	This study
mtr-R2	2081	TTACCCAGTAAAACTTGCAAACCAACCT AAGTTTGCCCCAATTTGCGCCATGTAGGCT GGAGCTGCTTCG	Disruption cassette	This study
tnaA-R1-NTHi375	2147	CGTGTTAGATTCGATTACTCCACC	Disruption cassette	This study
tnaB-R1-NTHi375	2163	GCCAAATTGACGATTTAATAACGTAATG	Disruption cassette	This study
tnaB-F2	2187	TGCTTAACAATGATAGTCATGACATTG	Disruption cassette	This study
tnaAB-F2-NTHi375	2150	TTCTTGCAATGTTTGGCTGCTTAC	Disruption cassette	This study
mtr-qPCR-F1	2192	GCGGAGGCATTACACAGAAT	qRT-PCR	This study
mtr-qPCR-R1	2193	CCACCATCCCGACAATTAAC	qRT-PCR	This study
F1_qPCR_*trpA*	2246	GGTGTTGATGCTGTGCTTGT	qRT-PCR	This study
R1_qPCR_*trpA*	2247	TGCGTGAGCTTGATTTTCAG	qRT-PCR	This study
tnaB-qPCR-F1	2190	TGGCGTAGGATTAGGCGTAT	qRT-PCR	This study
tnaB-qPCR-R1	2191	TTGCGGGATTTATAGGCAAG	qRT-PCR	This study
F2_qPCR_*sdaA*	2315	ATTATTCGATTGGGGGTGGT	qRT-PCR	This study
R2_qPCR_*sdaA*	2316	TGCGATTTCATTTTCCAACA	qRT-PCR	This study
gyrA-qPCR-F2	1078	ATATGTTGGTTGATGGGCAAGG	qRT-PCR	(7)
gyrA-qPCR-R2	1079	GGCGAGAAATTGACGGTTTCT	qRT-PCR	(7)
F-*AhR*-(m)-qPCR	2574	GAGCACAAATCAGAGACTGG	qRT-PCR	(8)
R-*AhR*-(m)-qPCR	2575	TGGAGGAAGCATAGAAGACC	qRT-PCR	(8)
F-*Socs-2*-(m)-qPCR	2572	CTCATCTCCCATGACCCTGC	qRT-PCR	This study
R-*Socs-2*-(m)-qPCR	2573	CCAGCTGACGTCTTAACGGA	qRT-PCR	This study
IL6m-qPCR-F	2140	AATCGTGGAAATGAGAAAAGAGTTGT	qRT-PCR	This study
IL6m-qPCR-R	2141	TCTCTGAAGGACTCTGGCTTTG	qRT-PCR	This study
m-*IL1β* qPCR F	1745	CAACCAACAAGTGATATTCTCCATG	qRT-PCR	This study
m-*IL1β* qPCR R	1746	GATCCACACTCTCCAGCTGCA	qRT-PCR	This study
KC-F	1404	GACAGACTGCTCTGATGGCA	qRT-PCR	(9)
KC-R	1405	TGCACTTCTTTTCGCACAAC	qRT-PCR	(9)
m*TNFα*-F	1592	AGGCACTCCCCCAAAAGATG	qRT-PCR	(9)
m*TNFα*-R	1593	GCTCCTCCACTTGGTGGTTT	qRT-PCR	(9)
m*Ido1*_qPCR_F	2325	GACTGAGAGGACACAGGTTACAG	qRT-PCR	This study
m*Ido1*_qPCR_R	2326	CCCACCAGGAAATGAGAACAGA	qRT-PCR	This study
m*GAPDH*-F	1430	CCCACTAACATCAAATGGGG	qRT-PCR	(10)
m*GAPDH*-R	1431	CCTTCCACAATGCCAAAGTT	qRT-PCR	(10)
P*sdaC*_Fw_SphI	2294	GGGCATGCCGCTGAAAATTACCGCACTTTT TATAA	Transcriptional analysis	This study
P*sdaC*_Rv_SpeI	2295	GGACTAGTCATAGTTTGCTCCAAAGCATAA ATTAC	Transcriptional analysis	This study
GFPend-AscI	2296	GGCGCGCCTTATTTGTATAGTTCATCCATGC CAT	Transcriptional analysis	This study
P*mtr*_GFP_Fw_SphI	2297	GGGCATGCATCGATATAAATATATTGCACT TTCCTTGCGTACTATTATAATAGTGCAAATT TATTTTAGAGGCATTTTATGACTAGTAAAG GAAAAGAACTTTTC	Transcriptional analysis	This study
GFP_Rv_BamHI	2400	GGGGATCCCCTTATTTGTATAGTTCATCCAT GCCATG	Transcriptional analysis	This study
GFP_Fw_SphI_TT	2468	GGGGCATGCCTGGATTCTCACCAATAAAAA ACGCCCGGCGGCAACCG AGCGTTCTGAACAAATCCAGATGGAGTTCT GAGGTCATTACTGGATCTATCAACAGGAGT	Transcriptional analysis	This study

		CCAAGATGACTAGTAAAGGAAAAGAACTTTTC		
RT_sdaA_Fw_1	2595	GTAGTGATAACGGCTTAATGC	Transcriptional analysis	This study
RT_sdaA_Fw_3	2597	AGGATTAACTTGCGACCCTG	Transcriptional analysis	This study
RT_mtr_Fw_1	2598	CAAATTGGGGCAAACTTAGGTTG	Transcriptional analysis	This study
RT_mtr_Fw_4	2601	CGATAGCTTATTGGGCAGAAC	Transcriptional analysis	This study
GFP_uni_Fw	2609	TCACTGGAGTTGTCCCAATTC	Transcriptional analysis	This study
RT_Cherry_Fw1	2610	AGGGCCGCCACTCCACCGGCG	Transcriptional analysis	This study

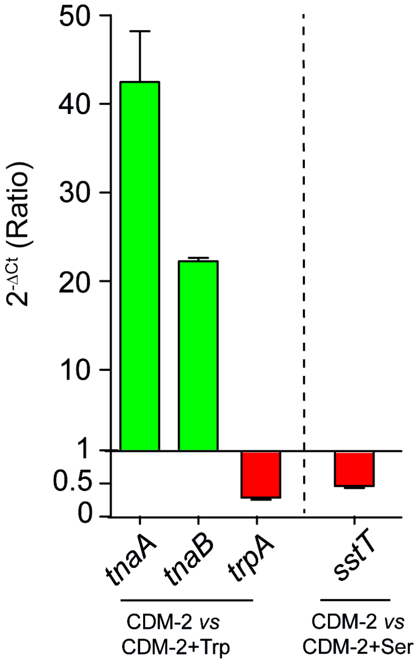

Figure S1. Effect of tryptophan supplementation on *H. influenzae* 375 strain gene expression. Validation of selected up- (green) and down-regulated (red) *H. influenzae* genes under CDM-2 growth in the absence or presence of tryptophan or serine. Bacterial cultures were grown in CDM-2, CDM-2+Trp or CDM-2+Ser and collected in exponential phase. Purified RNA was used to determine the ratio of bacterial gene expression by qRT-PCR. Data are shown as mean ± SD.

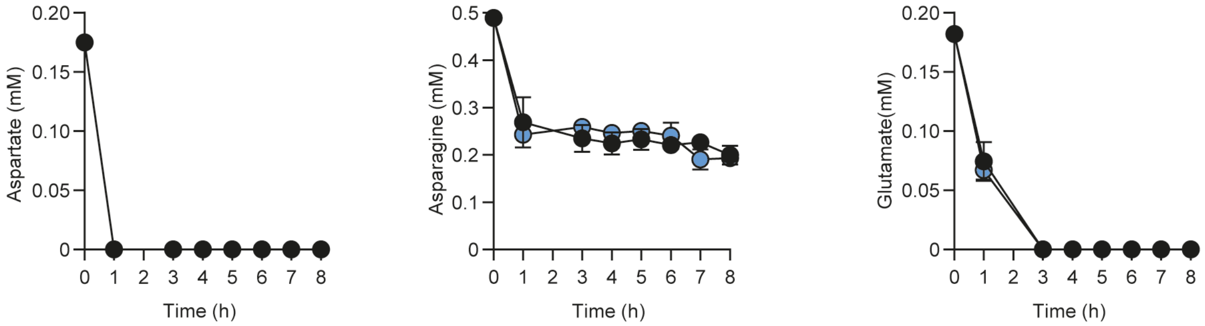

Figure S2. Amino acid consumption by NTHi375 during growth in CDM-2 in the absence/presence of tryptophan. Amino acid quantification in bacterial culture supernatants over time. NTHi375 was grown in CDM-2, in the absence (black) or presence (blue) of Trp. Strains were grown in sterile 250 mL flasks with 25 mL CDM-2 and shaking and 1 mL of supernatants were collected every hour for 8 h. Aspartate, asparagine and glutamate consumption was quantified.

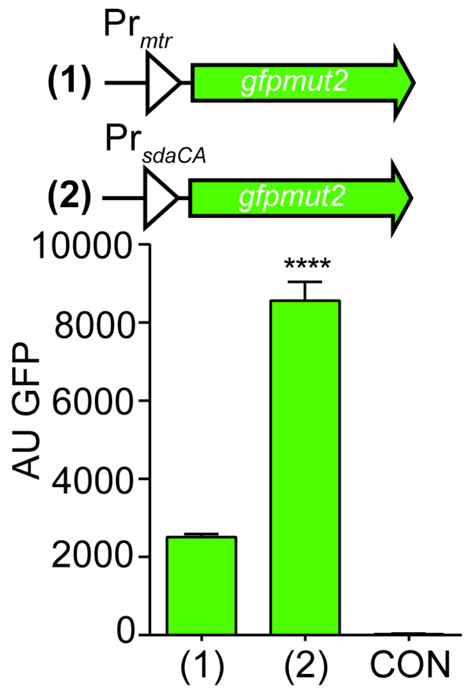

Figure S3. Definition of NTHi375 *mtr* and *sdaCA* promoter regions. Schematic representation of transcriptional fusions, cloned into pTBH plasmids, and introduced in the RdKW20 heterologous strain. RdKW20 was used as negative control (CON). Bacteria were grown on sHTM agar with Erm_{11} for 16 h. Then, two to five colonies were inoculated in 10 mL CDM-2 with Erm_{11}, and incubated for 12 h with shaking (100 r.p.m). Cultures were then diluted to OD_{600}=0.07 in 15 mL of CDM-2 with Erm_{11} and incubated in 50 mL flasks with shaking (200 r.p.m.). After 8 h, 200 µL aliquots of stationary phase grown cultures (OD_{600}=1.3) were transferred to 96-well plates (Nunc Optical Bottom plates with opaque polystyrene, Fisher Scientific, 165305) for fluorescence quantification in a SynergyH1 (BioteK) microplate reader. Fluorescence signal was quantified using a monochromator-based setting with specific excitation and emission wavelengths: green fluorescence protein (GFP) at 485/515 nm. Each fluorescence signal was corrected to its respective blank values (CDM-2). RdKW20 was used as a negative control. Statistical comparisons were performed using unpaired *t* test. Data are shown as mean ± SD (****P< 0.0001).

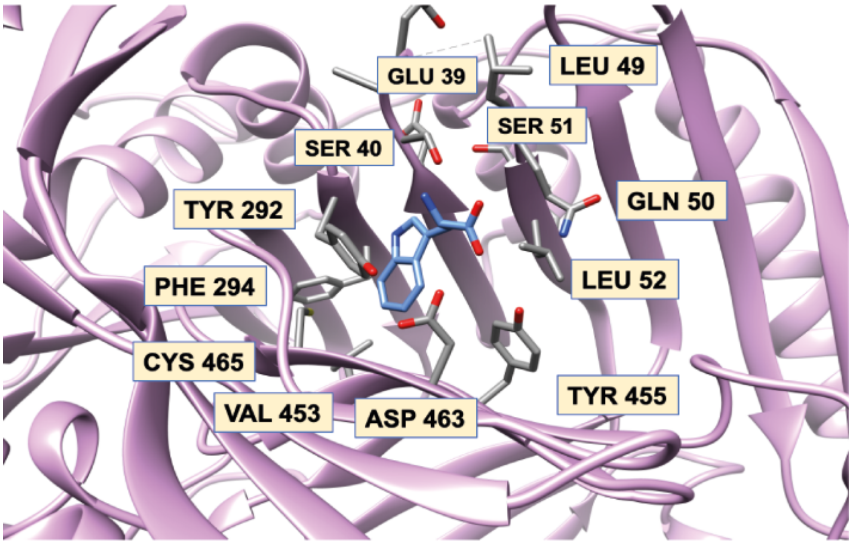

Figure S4. Prediction of TrpE$_{NTHi375}$ structure. TrpE crystal structure from *S. marcescens* (PDB ID 17IS, pink ribbon). Experimental L-tryptophan inhibitor binding site in *S. marcescens* from the 2.40 Å crystallographic structure.

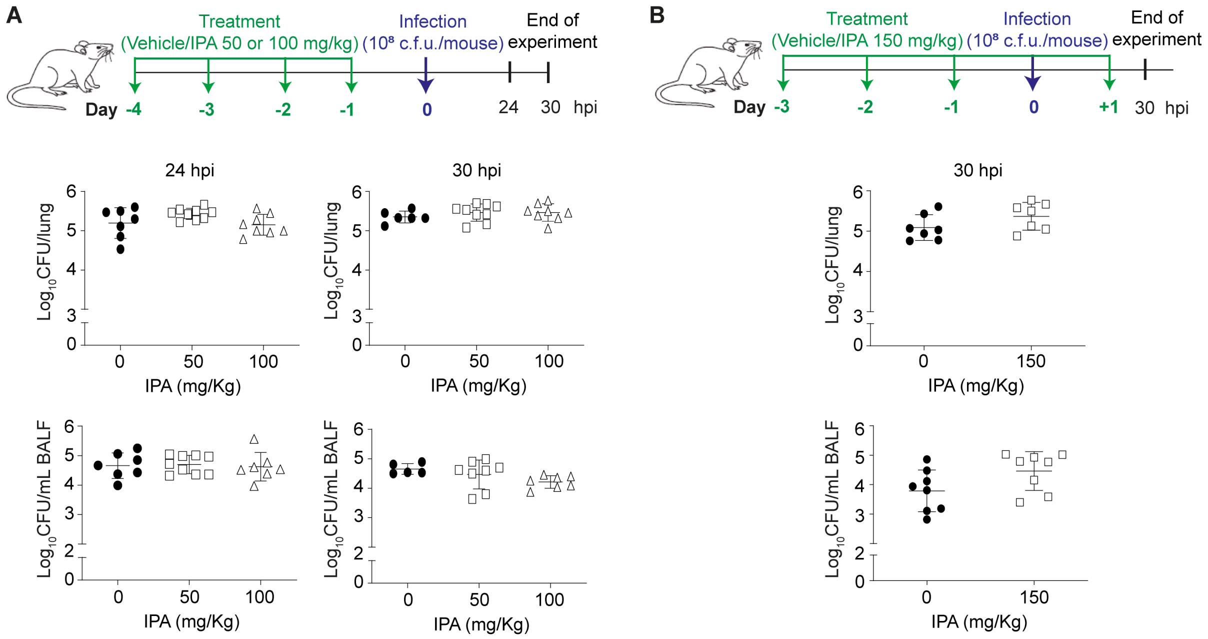

Figure S5. Effects of IPA administration on NTHi375 murine respiratory infection. CD1 mice were intranasally infected with NTHi375 WT strain (1x10^8 CFU per mouse). Bacterial counts were determined at 24 hpi or 30 hpi in lung (log_{10} CFU/lung) and BALF (log_{10} CFU/mL BALF) samples. Two regimens were used: **(A)** oral IPA (50 or 100 mg/kg), daily administrations during 4 days prior to infection, and **(B)** oral IPA (150 mg/kg), daily administrations during 3 days prior to- and 1 day post-infection. Results are shown as mean ± SD. Significant differences in terms of bacterial counts between control- and treated animals were not observed. Statistical comparisons of means were performed by one-way ANOVA and Dunnett's multiple comparisons test **(A)**, or by unpaired *t*-test **(B)**.

References

1. Bouchet V, Hood DW, Li J, Brisson JR, Randle GA, Martin A, et al. Host-derived sialic acid is incorporated into *Haemophilus influenzae* lipopolysaccharide and is a major virulence factor in experimental otitis media. Proc Natl Acad Sci USA. 2003 July 22;100(15):8898–903.
2. Fernández-Calvet A, Euba B, Gil-Campillo C, Catalan-Moreno A, Moleres J, Martí S, et al. Phase variation in HMW1A controls a phenotypic switch in *Haemophilus influenzae* associated with pathoadaptation during persistent infection. mBio. 2021;12(3):e00789-21.
3. Fleischmann RD, Adams MD, White O, et al. Whole-genome random sequencing and assembly of *Haemophilus influenzae* Rd. Science. 1995 Jul 28;269(5223):496-512.
4. Allen S, Zaleski A, Johnston JW, Gibson BW, Apicella MA. Novel sialic acid transporter of *Haemophilus influenzae*. Infect Immun. 2005;73(9):5291–300.
5. Tracy E, Ye F, Baker BD, Munson RS. Construction of non-polar mutants in *Haemophilus influenzae* using FLP recombinase technology. BMC Mol Biol. 2008;9:101.
6. Rapún-Araiz B, Sorzabal-Bellido I, Asensio-López J, Lázaro-Díez M, Ariz M, Sobejano de la Merced C, et al. *In vitro* modeling of polyclonal infection dynamics within the human airways by *Haemophilus influenzae* differential fluorescent labeling. Microbiol Spectr. 2023;11(6):e00993-23.
7. Moleres J, Fernández-Calvet A, Ehrlich RL, Martí S, Pérez-Regidor L, Euba B, et al. Antagonistic pleiotropy in the bifunctional surface protein FadL (OmpP1) during adaptation of *Haemophilus influenzae* to chronic lung infection associated with chronic obstructive pulmonary disease. mBio. 2018;9(5):e01176-18.
8. Zhao C, Bao L, Qiu M, Feng L, Chen L, Liu Z, et al. Dietary tryptophan-mediated aryl hydrocarbon receptor activation by the gut microbiota alleviates *Escherichia coli*-induced endometritis in mice. Microbiol Spectr. 2022;10(4):e00811-22.
9. Euba B, López-López N, Rodríguez-Arce I, Fernández-Calvet A, Barberán M, Caturla N, et al. Resveratrol therapeutics combines both antimicrobial and immunomodulatory properties against respiratory infection by nontypeable *Haemophilus influenzae*. Sci Rep. 2017 Oct 16;7:12860.
10. Regueiro V, Moranta D, Frank CG, Larrarte E, Margareto J, March C, et al. *Klebsiella pneumoniae* subverts the activation of inflammatory responses in a NOD1-dependent manner. Cellular Microbiology. 2011;13(1):135–53.
